# Development and validation of the puppy blues scale measuring temporary affective disturbance resembling baby blues

**DOI:** 10.1038/s44184-024-00072-z

**Published:** 2024-06-07

**Authors:** Aada Ståhl, Milla Salonen, Emma Hakanen, Salla Mikkola, Sini Sulkama, Jari Lahti, Hannes Lohi

**Affiliations:** 1 Department of Psychology and Logopedics, University of Helsinki, Helsinki, Finland; 2 Department of Veterinary Biosciences, University of Helsinki, Helsinki, Finland; 3 Department of Medical and Clinical Genetics, University of Helsinki, Helsinki, Finland; 4 Folkhälsan Research Center, Helsinki, Finland; 5 Department of Biology, University of Turku, Turku, Finland

**Keywords:** Human behaviour, Psychiatric disorders

## Abstract

It has been described that many puppy owners experience a state called *puppy blues* involving stress, worry, anxiety, strain, frustration, or regret. While puppy blues is a commonly used term among dog owners, the term is nearly nonexistent in scientific literature. In turn, analogous phenomenon, postpartum affective disturbance of infant caregivers, is well described in the literature. This study aimed to develop and validate the first questionnaire to evaluate puppy blues. The methodology involved generating scale items based on a qualitative review of 135 pilot survey responses from people who had experienced distress during the puppy period, conducting exploratory factor analysis for the final scale items from a dataset of 1801 answers from Finnish dog owners (92% women), and collecting test-retest data from 265 individuals to assess the consistency of the measurement of items and factor structure across time. In addition, we collected an independent sample of 326 owners of 1–2-year-old dogs who answered the survey both regarding puppy period and current moment. The results indicate that the scale is a valid and reliable tool for measuring dog owners’ negative experiences and feelings related to puppyhood. We discovered three factors that describe different aspects of puppy blues: Frustration, Anxiety, and Weariness, which accounted for a significant proportion of the variance in puppy blues. The study demonstrated good internal consistency and consistency across two independent samples for the three identified factors. The test-retest reliability of the factors was good. Responses for the current timeframe compared to puppyhood experiences revealed significantly lower current scores across all factors for the current period, validating that the scale captures distress during puppyhood that diminishes over time. Interestingly, we found a fading affect bias where recollections of the experiences in the puppy period became more positive with time. Our findings shed light on the characteristics of puppy blues and provide a useful retrospective tool for measuring it.

## Introduction

The emotional bond between humans and dogs can support the well-being of both parties^1–5^. However, like any relationship, there may be dysfunctional elements and caring for a dog can be a significant source of stress for the caregiver^6–8^. Caring for and raising a puppy is generally a happy event but can be stressful and burdensome for many people and evoke negative thoughts and feelings towards oneself and the puppy. Drawing from two recent qualitative studies, emerging evidence suggests that puppy owners may undergo “puppy blues”, encompassing feelings of emotional strain, anxiety about responsibility, and challenges in adapting to the changes brought by a new puppy^9,10^. As many dog owners consider their dogs to be a part of the family^11,12^, owners of puppies may encounter similar challenges and outcomes found in parents of infants. The term puppy blues is commonly used among dog owners^10^ and may be comparable to *baby blues*, which refers to a parental short-lasting dysphoric condition that can occur after childbirth and is characterized by symptoms such as anxiety, irritability or emotional lability, and tearfulness^13,14^. Both baby blues and puppy blues describe brief dysphoric episodes with symptoms resembling depression and anxiety that can occur after a major life change. In some cases, baby blues can develop into postpartum depression (PPD), a more severe and long-lasting diagnosable condition^14^. Similarly, it is possible that puppy blues can develop into a more severe state and may even lead to animal relinquishment if left unaddressed.

Research has already revealed that some forms of psychological distress observed in human relationships have analogies in human-canine relationships. In the same way that adult and infant-mother attachment can be characterized by insecure attachment styles, the bond between humans and their companion animals can also be described using the same dimensions^15,16^. Additionally, caring for an ill dog can be similarly stressful as caring for an ill person^6–8^. Studies on mental health have shown that parents of children with psychiatric disorders often experience adverse psychological effects^17,18^. Similarly, caring for a dog with unwanted behavior can be taxing for the owner^19,20^. Following this line of reasoning, there may be other analogous phenomena in the human-canine bond yet to be uncovered, such as the potential similarities between puppy blues and postpartum affective disturbance, encompassing the milder form known as baby blues and the more severe form known as PPD.

While there is currently limited knowledge about puppy blues, extensive research has been conducted on PPD. Many psychological factors can heighten the risk of developing PPD^21^. Risk factors for PPD can be divided into three main categories: psychosocial, clinical, and personality or temperamental features^21^. Certain personality traits are associated with increased vulnerability to PPD. Among these, the evidence is most robust for neuroticism^21,22^. Similar predisposing factors may also exist for puppy blues. In addition to exploring the predisposing factors, it is interesting to consider other potential parallels between puppy blues and postpartum depression. Similar to how depressed parents may exhibit behaviors that can impact their infant’s development^23^, it is possible that severe dysphoric symptoms in puppy caregivers also lead to caregiver behaviors that affect the puppy’s future behavior or well-being. Understanding the effects of puppy blues on the quality of care received by the puppy is particularly crucial as environmental factors can significantly influence a dog’s development during the sensitive period of socialization, typically occurring between 3 and 16 weeks old^24^. Further research on this topic is crucial for promoting healthy and positive interactions between puppies and their caregivers.

While the individual and public health impacts of postpartum affective disturbance are relatively well-established, dysphoric episodes have been only minimally explored in individuals who provide care for a puppy. The term “puppy blues” has gained recognition in scientific literature only since 2023 through two qualitative studies, despite its prevalent use among dog owners^9,10^. Although tools have been developed to assess postpartum affective disturbance, to our knowledge, there is currently no tool for detecting and examining this problem in interspecies relations, leading to a limited understanding of the characteristics of the puppy blues. Moving forward, it is essential to expand our understanding of the impact of caring for a new companion animal on mental health. By addressing this gap in the literature, we can provide better support and resources to those struggling with caring for a new puppy and improve the well-being of both the dog and the owner. To better understand the phenomenon of puppy blues, we need a tool to measure it. Self-report questionnaires are common in measuring aspects of mental health, and several different prospective and retrospective questionnaires have been developed to measure PPD (e.g., Edinburgh Postnatal Depression Scale, EPDS^25^). A similar self-report scale for puppy owners to measure dysphoric state following puppy adoption would be useful but the scale would need to be simple to complete and have good reliability and validity. Our specific objectives were (a) to develop a self-report questionnaire to evaluate puppy blues and (b) to assess the validity and reliability of the scale by examining internal consistency, test-retest reliability, predictive validity, cross-validity, construct validity, and convergent validity.

## Methods

### Item development

Since we could not rely on literature review and assessment of existing scales (deductive “top-down” methods) in generating the scale items, as there were no prior studies or scales on puppy blues at the time, we used the inductive “bottom-up” method by generating items from the responses of individuals to a pilot survey. The participants were recruited through social media from June 2022 to July 2022. Participants were invited to fill out the pilot survey if they had experienced negative feelings during their dog’s puppyhood. They were informed that their responses would be used to develop a survey to measure negative emotions and mental strain associated with the puppy period. The pilot survey included three open-ended questions: 1) “Freely discuss the emotional burden associated with the puppy period and the negative feelings and thoughts you had regarding the puppy and puppyhood”, 2) “How long did these feelings approximately last?”, and 3) “Did you take any actions to cope with or alleviate these feelings?”.

We qualitatively analyzed 136 individual responses to the first question, identifying recurring adjectives and domains in the texts, and calculating their frequencies. Five responses were excluded: one didn’t pertain to the experiences of a puppy owner but rather of a breeder before the weaning age; one was empty; two portrayed only positive experiences; and one described the burden related to a simultaneous challenging life event instead of the puppy phase. Key themes emerged in the remaining 131 responses, with the most common being exhaustion, fatigue or sleep problems (45%), feelings of inadequacy as a dog owner (39%), anxiety about the puppy taking up time and attention (36%), concerns about raising the puppy “correctly” (30%), feelings of regret about getting a puppy (24%), difficulty in forming an emotional bond with the puppy (19%), disappointment with the puppy phase not meeting expectations (19%), feelings of anger and irritability towards the puppy (19%), worry for the puppy’s well-being (15%), and considering giving the puppy away (11%). We wanted to focus on psychological symptoms and symptoms that are not dependent on family composition or socioeconomic status. Thus, we decided to exclude domains of physical symptoms of low appetite and nausea (5%), worry about the expenses caused by the puppy’s damages (3%), family conflicts (1%), and concern about how the puppy will get along with existing dogs in the family (5%). Additionally, we chose not to include the element of crying (9%) in the final item pool, considering that the item asking about crying in the EPDS postpartum depression scale is recognized for being more aligned with the female experience of distress, given its infrequent endorsement by men^26^. Fifteen scale items were generated (Table 1).

**Table 1:** Measurement instrument for puppy blues symptoms among dog owners

*During your dog’s puppyhood, how often…*	Almost never or never	Sometimes	Fairly often	Almost all the time	I don’t know
1. …did the puppy and taking care of the puppy feel more difficult than you had expected?	0	1	2	3	-
2. …were you worried about the well-being of the puppy?	0	1	2	3	-
3. …did you feel irritation/anger towards the puppy?	0	1	2	3	-
4. …did you feel inadequate and incompetent as a dog owner?	0	1	2	3	-
5. …did you think about giving away the puppy?	0	1	2	3	-
6. …did you worry that you would “ruin” the puppy with your actions?	0	1	2	3	-
7. …did building an emotional bond and connection with the puppy seem difficult?	0	1	2	3	-
8. …did you feel exhausted?	0	1	2	3	-
9. …did you have trouble sleeping, even when the puppy slept well?	0	1	2	3	-
10. …were you anxious that the puppy was taking up all your attention and time?	0	1	2	3	-
12. …did you feel bad conscience and guilt towards yourself as a dog owner when things didn’t go as you expected?	0	1	2	3	-
13. …did you worry about caring for and raising the puppy “correctly”?	0	1	2	3	-
14. …did everything you do feel like an effort?	0	1	2	3	-
15. …did you question or regret your choice to get a puppy?	0	1	2	3	-
REMOVED ITEMS:					
11. …did you watch over the puppy in case something bad would happen to the puppy?^1^	0	1	2	3	-
16. Overall, when you think about the time when you felt the most distressed during your dog’s puppyhood, how burdened did you feel?					

Not at all			Moderately			Extremely
1	2	3	4	5	6	7

^1^This item was removed based on inter-factor loadings.

### Scale development: sampling and survey administration

To obtain a comprehensive population of dog owners, we sent online survey invitations to 2296 individuals who had previously participated in our studies on dog personality, behavior, owner well-being, personality, and attachment style^27,28^. These participants were initially recruited through social media and included a diverse range of Finnish respondents from different age groups who owned over 200 different dog breeds and various numbers of dogs. Data were collected using REDCap electronic data capture tools hosted at the University of Helsinki^29,30^. Responses were collected from December 2022 through February 2023. In total, we received 1801 responses. Before analyses, we excluded two cases with more than five missing items (i.e., answered with the option “I don’t know”).

During the last months of the data collection, we selected a set of dog owners who had answered the survey 1–4 months prior and sent them a request to participate in the test-retest reliability study. These owners were requested to answer the questionnaire sections again. The final test-retest dataset included responses from 259 owners encompassing their experiences of a total of 265 dogs.

**Fig. 1:**
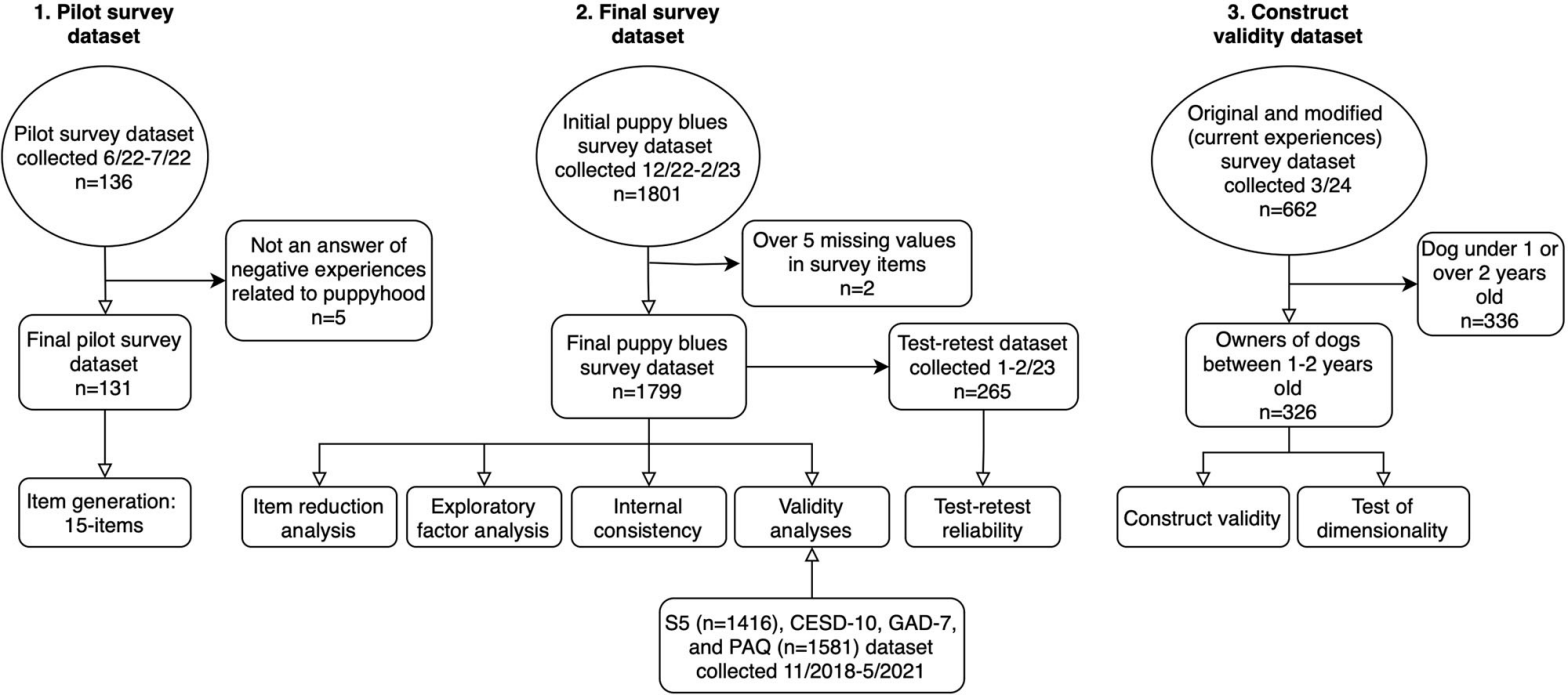
Flow chart of the sample size and analyses.

To explore if the survey captures transient burden during the puppy phase that diminishes as dog age, we collected separate data from 326 owners of young dogs (1–2 years old) in March 2024. We asked them to respond to the survey twice; first regarding their experiences during the puppy period and then regarding their current experiences with a modified version of the survey, in which we modified the survey into the present tense and replaced the words “puppy” with “dog”. The compilation of all data and analyses performed on various datasets is illustrated in Fig. 1.

### Scale development: extraction of factors

To ensure the survey only included concise and internally consistent items, we conducted an item reduction analysis. This involved estimating inter-item and item-total correlations (Supplementary Tables 1 and 2), demonstrating that all items correlated adequately with other items and well with the total score. Inter-item correlations were estimated using Pearson correlations and visually represented with the *corrgram*^31^ package. Item-total correlations were calculated using the *multilevel*^32^ package. To enhance the accuracy of item-total correlations, we utilized the *psych*^33^ package and its *alpha* function to compute corrected item-total correlations.

We performed exploratory factor analysis (EFA) to reduce the questionnaire items into fewer meaningful domains. Before factor analysis, we tested the suitability of our dataset for factor analysis with the Kaiser-Meyer-Olkin test for sampling adequacy from the package *psych*^33^. We conducted the factor analysis with the package *psych*^33^. We used polychoric correlation matrices and conducted the factor analyses with oblimin rotation from the *GPArotation*^34^ package and mean imputation. We opted for oblimin rotation due to our expectation that the factors would be correlated with each other. We evaluated the number of factors to be extracted with the scree test and Velicer’s minimum average partial (MAP) test. Furthermore, we assessed the quality of the factor structure by extracting all possible structures (Goldberg’s hierarchical tree) starting from one factor up to two factors more than recommended by the scree test. We evaluated the conceptual interpretability of the competing factor structures, as well as compared the root mean square error of approximation (RMSEA) and the Tucker-Lewis index between these structures. We observed several cross-loadings and factors with less than three non-cross-loading items in the most suitable model with all 15 items. To improve the model fit, we removed one item related to monitoring and guarding the puppy in case something bad could happen (which we named “wary”). This item did not load uniquely on individual factors but loaded onto all factors. Moreover, the item-total correlation of the item was the lowest (.34) among all the items (Supplementary Table 2).

### Scale evaluation: test of dimensionality

To assess the consistency of the measurement of items, factors, and their function across two independent samples, we tested dimensionality using confirmatory factor analysis (CFA). We performed CFA with the package *lavaan*^35^ to the separate data set of 326 owners of young dogs. We estimated several models, including one-, two-, three-, and four-factor models, and a confirmatory bifactor model with a general factor and sub-scales. We also estimated a revised three-factor model in which items with low standardized factor loadings ( < .30) were excluded. We fitted the models with all the original 15 items and with the exclusion of the item “wary”. We compared the root mean square error of approximation (RMSEA), comparative fit index, and the Tucker-Lewis index between these structures.

### Scale evaluation: reliability

We assessed the reliability of the scale by calculating Cronbach’s Alpha and Guttman’s Lambda 6 with the package *psych*^33^ for all factors. To assess test-retest reliability, we used the package *psych*^33^ for calculating the Pearson and Intraclass correlations. These correlations were computed on responses from 265 participants who completed the survey twice, comparing their answers between the first and second time. The intraclass correlation coefficient (ICC) was computed using the ‘two-way random effects, absolute agreement, single rater/measurement’ model (ICC 2,1)^36,37^. These correlations between first and second response to the questionnaire were calculated for all items and extracted factors.

### Scale evaluation: validity

We assessed predictive validity, cross-validity, construct validity, and convergent validity. Before validity analyses, we extracted the factor scores for all individual responses with the package *psych*^33^, using a correlation-preserving estimation method (“tenBerge”) for multifactorial structures. These factor scores were used in subsequent validity analyses. We assessed the predictive validity of the scale by examining the association between the factor scores and the subjective burden score (Table 1, Item 16; ”Overall, when you think about the time when you felt the most distressed during your dog’s puppyhood, how burdened did you feel?”) with polyserial correlations using the package *polycor*^38^ and boxplots using the package *ggplot2*^39^ and *ggpubr*^40^. We assessed the cross-validity by randomly splitting the data into two samples using the *caret* package^41^. A 2-fold cross-validation strategy was used to conduct the factor analysis on the training sample and subsequently test the factor solution on the test sample. We also explored whether there were any trends in the factor scores regarding how much time had elapsed since puppyhood. In other words, we explored whether dog owners who had a longer time since their dog’s puppyhood evaluated the period more positively, similar to the fading affect bias^42,43^. To explore this relationship, we plotted each factor score against the amount of time that had passed since the puppy period on scatter plots using the package *ggplot2*^39^.

To examine construct validity, we also assessed whether the scale measures distress specifically during the puppy phase, decreasing as the dog matures into adulthood. To explore this, we examined whether dog owners receive lower scores on the survey related to experiences when the dog is 1–2-year old, by comparing the responses of 326 owners of young dogs regarding their experiences during the puppy period and their current experiences. We computed factor scores for both survey responses (puppyhood and current experiences) using the *predict* function from the *psych* package^33^. Paired samples t-tests were conducted to compare scores for each factor during the puppy phase and the current period. Additionally, we calculated a paired samples t-test for the subjective burden score during the puppy phase and the current moment. We visualized the difference in factor scores with boxplots using the package *ggplot2*^39^ and *ggpubr*^40^.

We evaluated the convergent validity by hypothesis testing. Firstly, based on the literature on risk factors for PPD^21^, we hypothesized that the owner’s personality trait of neuroticism would positively correlate with higher puppy blues scores. Secondly, we hypothesized that the anxiety factor within the context of puppy blues might exhibit qualitative parallels with pet attachment anxiety, while the frustration factor could potentially manifest parallels with pet attachment-related avoidance. As a result of this qualitative assessment of shared phenomena, we expected to observe positive correlations among these factors. Thirdly, we hypothesized that the puppy blues scores are positively correlated with later anxiety and depression symptoms. This proposition is grounded in the notion that there could be shared risk factors between puppy blues and these subsequent mental health outcomes. Drawing from the literature on PPD, there is evidence to suggest that a lifetime history of depression and anxiety is associated with an increased risk of developing PPD^44,45^.

For testing these hypotheses, we utilized previously gathered data from a survey on owner personality and well-being^28^, which had been completed by the participants in this study. The data collection occurred from November 2018 to May 2021 through online surveys. Owner neuroticism was assessed using the 64-item Short Five Inventory (S5)^46^. Attachment styles were evaluated using the 26-item Pet Attachment Questionnaire (PAQ)^15^. Anxiety symptoms were measured using the 7-item Generalized Anxiety Disorder Scale (GAD-7)^47^, and depressive symptoms were assessed using the 10-item Center of Epidemiologic Studies Depression Scale (CESD-10)^48^. To calculate the validity coefficients, we employed Pearson correlations using the package *psych*^33^. We corrected all p-values for false discovery rate (FDR) to decrease the probability of type I error. The significance cut-off *p*-value was set at *p* < 0.001.

All analysis and data handling were conducted on R version 4.3.2^49^. Data editing was done with the package *dplyr*^50^.

## Results

### Descriptive statistics

We collected 136 individual responses with the pilot survey. In total, the final dataset, compiled with the new survey instrument developed based on the pilot survey answers, comprised responses from owners of 1801 dogs. Overall, 1572 dog owners answered the survey, with an average of 1.3 dogs. Out of the respondents, 1448 identified as women, 56 as men, 12 as other or preferred not to disclose their gender, and the gender of 56 respondents was unknown. The sample included owners of dogs in 224 breeds and breed variants. Of the dogs, 49.5% were females and 50.5% were males. Time from the dog’s birth varied between 2.62 and 20.23 years, with an average of 7.96 years since the dog’s birth (sd 3.32 years). Overall, 45.1% of the respondents reported experiencing significant negative feelings during their dog’s puppyhood. Among those who encountered significant negative feelings, 20.3% reported that these feelings persisted for less than a month, 31.0% reported a duration of 1–5 months, 29.5% experienced feelings lasting from half a year to a year, and 19.3% reported that these feelings endured for over a year. The test-retest reliability dataset included 259 dog owners who responded for a total of 265 dogs, and the time between the two answers varied between 50 and 95 days, with mean time difference of 80 days (sd 6 days). Within the separate dataset of owners of 1–2-year-old dogs, 32.8% of the responses corresponded to dogs aged 12–15 months, with 22.1% to dog aged 16–18 months, 20.3% to dogs aged 19–21 months, and 24.9% accounted for dogs aged 22–24 months.

### Item generation

Through careful review of the pilot survey responses, we identified the most frequently recurring symptoms in the responses and developed 15 scale items based on these symptoms. We also included questions about subjective severity of the burden (on a scale from 1 to 7), length of the symptoms, time period an individual waited to receive a puppy (the time from the first contact with a kennel or other place of acquisition to the dog’s arrival at home), coping methods (internal and external strategies that individuals use to manage distress; generated based on the responses to the pilot survey), and simultaneous stressful life events (adapted from LEC-5^51^) in the final survey to examine possible associations in future studies. The 15 questionnaire items and the burden severity item are shown in Table 1 and the other questions can be found in Supplementary Notes.

### Factor structure

The best factor structure in puppy blues scale items, examined using EFA, included three factors, which we named Frustration, Anxiety, and Weariness (Table 2). In refining the best factor structure, model fit was enhanced by removing one item related to monitoring the puppy in case something bad happened (Table 1, item 11). The three factors accounted for 72% of the variance in puppy blues. Anxiety accounted for the highest proportion of the variance (39%), followed by Frustration (34%) and Weariness (28%). There were significant positive correlations between Anxiety and Frustration (*r* = 0.59, *p* < 0.001), Anxiety and Weariness (*r* = 0.64, *p* < .001), as well as Frustration and Weariness (*r* = 0.64, *p* < 0.001).

**Table 2:** Item loadings in the exploratory factor analysis of the puppy blues questionnaire

Item no^a^	Item (abbreviated)	Anxiety	Frustration	Weariness
6	Fear of “ruining” the puppy	**0.92**	−0.04	−0.07
13	Worry about raising puppy correctly	**0.92**	−0.09	0.05
12	Guilt as a dog owner	**0.75**	0.16	0.02
4	Feeling of inadequacy	**0.68**	0.24	0.03
2	Concern for the puppy’s well-being	**0.46**	−0.12	0.24
5	Considering giving away the puppy	−0.01	**0.88**	0.00
15	Regret	0.03	**0.82**	0.06
3	Irritation towards the puppy	0.15	**0.61**	0.10
7	Challenges in forming an emotional bond	0.19	**0.48**	0.07
1	Difficulty in taking care of a puppy	0.16	**0.39**	**0.41**
14	Cumbersome	0.28	**0.34**	**0.39**
8	Exhaustion	−0.01	0.19	**0.79**
9	Sleep problem	0.03	−0.20	**0.79**
10	Anxiety about required attention and time	0.08	0.20	**0.58**

Loadings > 0.30 are in bold.

^a^Item order in the questionnaire.

### Test of dimensionality

The three-factor model (in which item 11 was removed) achieved satisfactory model fit in CFA for the separate data of owners of 1–2-year-old dogs suggesting that the three-factor structure identified by the EFA is consistent across two independent samples (Supplementary Table 5).

### Reliability and internal consistency

Internal consistency of the factors was good (Table 3). The mean Cronbach’s alpha for the factors was 0.82 and the mean Guttman’s Lambda 6 for the factors was .84. The factor-specific values can be found in Table 3.

The test-retest reliability of the factors was good (Table 3). The Pearson correlations between the two time points had a mean of .83. The Intraclass correlation between the two time points had a mean of 0.80. The factor-specific values can be found in Table 3. Test-retest reliability estimates of individual items can be found in Supplementary Table 3.

**Table 3:** Internal consistency and test-retest reliability of puppy blues factors

	Internal consistency		Test-retest reliability	
Factor	Cronbach’s Alpha	Guttman’s Lambda 6	Pearson correlation	Intraclass correlation
Frustration	0.81	0.83	0.88	0.85
Anxiety	0.86	0.86	0.81	0.80
Weariness	0.80	0.82	0.80	0.75
**Mean**	0.82	0.84	0.83	0.80

### Predictive validity

The results suggest that the scale has good predictive validity, as all factors demonstrated linear associations with the subjective burden score (Supplementary Fig. 1). Specifically, the Weariness factor showed a high correlation of 0.76, while Anxiety and Frustration also exhibited significant correlations of .69 and .63 respectively, with subjective burden. These associations were statistically significant (*p* < 0.0001, df = 1 797).

### Cross-validity

The results from the cross-validation procedure indicate that the factor structure is reliable and holds up well in the test sample (Supplementary Table 4). Specifically, the factor loadings for each variable in the EFA conducted for the training sample are highly consistent with those in the original sample. Furthermore, the confirmatory factor analysis results for the test sample indicate that the model fits the data well based on various fit indices (Supplementary Table 5).

### Construct validity

**Fig. 2:**
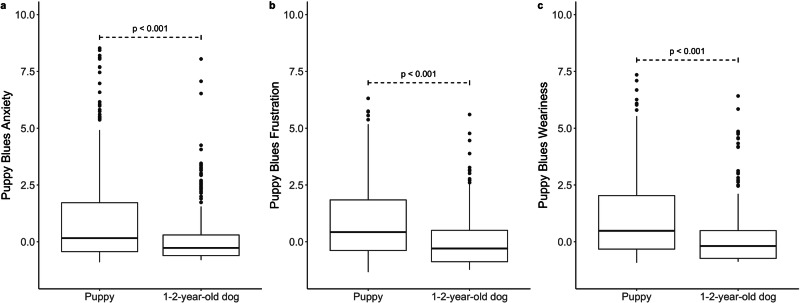
Box plots comparing responses related to puppyhood to present experiences in 1–2-year-old dog owners (*n* = 326). In the figure, the difference in factor scores is presented for (**a**) the anxiety factor, (**b**) the frustration factor, and (**c**) the Weariness factor, with the p-value indicating the significance of differences between groups calculated using a paired samples t-test.

The results reveal significant differences in the current experiences reported by owners of 1–2-year-old dogs compared to their recollections of experiences during the puppy period (Fig. 2). The scores for each factor were significantly lower related to the current moment compared to the responses recalling the puppy phase. The mean difference for anxiety factor was −0.88 (*t* = −9.26, df=319, *p* < 0.001), for frustration factor −0.84 (*t* = −12.45, df = 319, *p* < 0.001) and for weariness factor −0.88 (*t* = −10.66, df=319, *p* < 0.001). Anxiety factor score decreased or remained the same for 73.93% of the sample, frustration factor score decreased or remained the same for 78.83%, and weariness factor score decreased or remained the same for 76.69% (Supplementary Table 6). The mean difference for subjective burden was −0.89 (*t* = −8.49, df = 325, *p* < .001). These findings support the construct validity of the scale, indicating that the scale captures transient burden, being highest during the puppy phase and subsequently diminishing when dogs mature.

Our findings suggest a slight fading affect bias, i.e., a positive trend between the time that had elapsed since the puppy period and more positive recollections of the experiences (Supplementary Fig. 2). This supports the measure’s ability to capture experiences that are expected to be susceptible to the fading affect bias, thereby supporting the construct validity of the scale.

### Convergent validity

The results from hypothesis testing reveal significant positive correlations within the context of puppy blues and psychological factors, thus supporting the convergent validity of the puppy blues questionnaire (Table 4).

**Table 4:** Hypotheses formed to examine the convergent validity of the questionnaire, and their Pearson correlation coefficients, sample sizes, and p-values

Factor	Hypothesis	Pearson correlation	*n*	*p*-value
Anxiety	Owners scoring higher on neuroticism have higher puppy blues anxiety scores	0.36	1416	<0.001
	Owners with higher scores on the puppy blues anxiety factor have more anxious pet attachment style	0.31	1581	<0.001
	Puppy blues anxiety scores are positively correlated with later anxiety symptoms	0.32	1076	<0.001
	Puppy blues anxiety scores are positively correlated with later depression symptoms	0.30	1076	<0.001
Frustration	Owners scoring higher on neuroticism have higher puppy blues frustration scores	0.21	1416	<0.001
	Owners with higher scores on the puppy blues frustration factor have more avoidant pet attachment style	0.27	1581	<0.001
	Puppy blues frustration scores are positively correlated with later anxiety symptoms	0.15	1076	<0.001
	Puppy blues frustration scores are positively correlated with later depression symptoms	0.16	1076	<0.001
Weariness	Owners scoring higher on neuroticism have higher puppy blues weariness scores	0.26	1416	<0.001
	Puppy blues weariness scores are positively correlated with later anxiety symptoms	0.22	1076	<0.001
	Puppy blues weariness scores are positively correlated with later depression symptoms	0.23	1076	<0.001

All *p*-values were corrected for false discovery rate (FDR).

## Discussion

We created a scale to measure puppy blues, the dysphoric state of feeling distressed, worried, anxious, frustrated, irritated, strained, or regretful after bringing home a puppy. We conducted analyses to assess its reliability and validity. Our study demonstrates that this instrument is a reliable and valid tool for retrospectively evaluating symptoms of puppy blues. This is significant because, until now, no such instruments have been available to address this common issue, which may directly impact the well-being of the puppy and the caregiver.

We discovered that in dog owners, the new role and significant life changes that come with acquiring a puppy can trigger feelings of anxiety, which may manifest as exaggerated guilt and disproportionate worries. In addition, individuals may sometimes experience irritability or difficulty forming an emotional bond with the puppy as well as fatigue and sleep problems. Therefore, puppy blues resembles “baby blues”, as both conditions are characterized by comparable symptoms such as dysphoric mood, anxiety, insomnia, and irritability^13,14^. In postpartum affective disturbance, the sensitization of mood following childbirth exists on a continuum, with normal emotional sensitization on one end and psychological symptoms requiring external help on the other^14^. We discovered that puppy blues symptoms can also be characterized as continuous factors, with no to few symptoms on one end and many frequently occurring symptoms on the other. In our dataset, just under half of the respondents reported experiencing significant negative feelings during their dog’s puppyhood. The prevalence of distress seems to align with reported occurrences of baby blues, where it has been suggested that around 38% of women experience similar feelings after giving birth^52^. Among those who have the puppyhood of their dog fresh in memory, i.e., in the dataset of owners of 1–2-year-old dogs, 10.1% of owners reported feeling extremely burdened during the puppy period. Prevalence rates for more severe PPD vary^53^, but in a Finnish cohort study, the prevalence is reported to be 10.3%^54^.

We identified three separate but correlated factors: Frustration, Anxiety, and Weariness. The anxiety factor primarily focuses on self-doubt and feelings of inadequacy as a dog owner, as well as concerns about the puppy’s well-being and development. The frustration factor, on the other hand, measures the overall sense of dissatisfaction and emotional strain experienced due to the challenges and unexpected difficulties in caring for a puppy, including feelings of irritation, doubts about the bond with the puppy, perceiving tasks as demanding and questioning the decision to get a puppy. The third factor, Weariness, captures the strain associated with puppy care, including the challenges of the perceived difficulty of taking care of the puppy, exhaustion, sleep disturbances, anxiety regarding the time and attention required by the puppy, and the perception of heightened effort during the puppyhood phase. Identifying three factors contributing to the experience of puppy blues helps us understand the different aspects of this phenomenon. The high proportion of variance accounted for by anxiety suggests that it is a prominent aspect of puppy blues and may be a key area for intervention or support.

While nearly half of the participants acknowledged experiencing distress during their dog’s puppyhood, it is noteworthy to recognize that this phase appears relatively positive and non-burdensome for many people. Acquiring a puppy can yield positive effects on mental well-being. A study comparing pet owners and non-pet owners living alone found dog ownership to play a protective role against loneliness during the COVID-19 lockdown^55^. Longitudinal studies further support the notion that obtaining a dog can reduce loneliness^56,57^. On the other hand, it is crucial to note that experiencing puppy blues does not preclude the potential for obtaining positive effects from having a dog, such as protection against loneliness.

Two previous studies on puppy caregivers’ challenges align with our findings on puppy blues. In a study employing qualitative analysis of online discussions about dog behavior, puppy blues stemmed from two main components: the emotional pressure to handle tasks “correctly” and the exhaustion and fatigue linked to puppy care^10^. Notably, these components align with the factors identified in our study, specifically Anxiety and Weariness. Another qualitative study delved into the experiences of 19 Australian puppy owners, revealing narratives that not only emphasized positive aspects but also brought to light challenges faced by puppy owners, including struggles to manage conflicting demands on their time, concerns about the puppy’s health and safety, and feelings of regret^9^.

In addition, the identified aspects of puppy blues share several commonalities with the caregiver burden reported in studies involving owners of pets with unwanted behavior and illnesses. The frustration factor in puppy blues aligns with the emotions reported by owners of pets with unwanted behavior experiencing frustration, anger^19^, irritability and discomfort with the presence or behavior of the dog^20^. Feelings of frustration are also reported among owners of ill dogs^7^. The Anxiety factor also resonates with burden related to dog’s unwanted behavior, as these owners report feelings of guilt, embarrassment^19^, fear for the dog’s health or future and guilt for not doing enough^20^. This resemblance extends to owners of ill dogs, who similarly express feelings of guilt and worries about the future^6,7^. Lastly, the Weariness factor shares commonalities with experiences of caring for dogs with unwanted behavior involving extra time demands, limitations on movements^19^, weariness based on perceived negative changes in daily routines, tiredness, and perceived overload^20^. Owners of ill dogs also report additional care responsibilities and restrictions related to work and social life^7^.

Similarities can also be found in the factors of puppy blues and dimensions of an instrument measuring caregiving burden in caregivers of children with emotional problems, the Caregiver Strain Questionnaire (CGSQ). The CGSQ measures three dimensions of caregiver strain: objective caregiver strain relating to time and effort required to attend to the needs of the children; internalized subjective caregiver strain capturing negative feelings such as worry and guilt; and externalized subjective caregiver strain referring to negative feelings experienced by the caregiver toward the child, including anger, resentment, and relating poorly with the child^58^. Parallels can be observed between the puppy blues anxiety and the subjective internalized caregiver strain and with the puppy blues frustration and the externalized subjective caregiver strain. Thus, it seems that there are generalizable features of caregiver burden related to providing care to different care recipients, and this is the first time the phenomenon has been reported more broadly among caregivers of healthy dogs.

Our puppy blues scale exhibited good internal consistency (Cronbach’s alpha: .80-.86, considered “good” when between .80 and .89^59^) and excellent test-retest reliability (Intraclass correlation: .75-.85, considered “excellent” when between .75 and 1.00^59^). Despite lacking a direct comparative measure for puppy blues, our puppy blues scale demonstrates comparable reliability to other established measures. In comparing the validity of our puppy blues scale to the Pet Attachment Questionnaire (PAQ)^15^, which assesses attachment anxiety and avoidance in human-pet relationships, the internal consistency and temporal stability are similar: Cronbach’s alpha of PAQ factors were .86-.87 and the test-retest coefficients were .75 for attachment anxiety and .80 for attachment avoidance^15^. When comparing the puppy blues scale to the CGSQ, both scales demonstrate good internal consistency. In the CGSQ, the internal consistency for the factors in the original study is .92 for the objective strain, .74 for the externalized subjective strain subscale, and .86 for the internalized subjective strain subscale^58^. The test-retest reliability of the scale has been examined in two studies focusing on the burden of caregivers of children with neuropsychiatric disorders, showing reliabilities of .72^60^ and .89^61^ for the overall scale. In the latter study, factors’ test-retest reliabilities were also explored with intraclass correlations, with values of .92 for objective strain, .63 for externalized subjective strain, and .87 for internalized subjective strain^61^.

Dogs are puppies for only a brief period, and the timing of the puppy blues may vary individually. Therefore, our scale was designed and constructed based on retrospective recollections of the puppy blues, not as a scale for use during the experiences. We found evidence for the construct validity of our scale, as owners of young dogs rated their feelings of burden much lower in the time of answering compared to the puppyhood, meaning that the feelings of puppy blues are mostly transient. Interestingly, we found a slight positive trend between the time elapsed since the puppy period and more positive recollections of the experiences. Therefore, the accuracy of the recollections may have been influenced by memory biases or inaccuracies. People may have incorporated their long-term observations of their dog into their responses, which may have affected the results. However, it is important to note that the number of respondents who had a long time since their dog’s birth ( > 17 years) was very small, which naturally results in less variation in the values and makes it difficult to draw firm conclusions. Therefore, while our scatterplots provide some evidence for a potential time-related effect on recollections of the puppy period, the results should be interpreted cautiously. Detection of this trend can strengthen the scale’s construct validity, as it demonstrates the ability of the scale to capture phenomena that are expected to be influenced by this bias. Further analysis could consider the amount of time that has elapsed since the puppy period as an explanatory variable to better account for the fading effect bias.

Our study has several limitations that should be considered. Firstly, we were unable to test concurrent criterion validity or convergent validity due to the lack of an established “gold standard” or comparable scale for measuring the same construct. Future research could address this limitation by exploring the concurrent criterion validity if another suitable measure for assessing the puppy blues becomes available. Secondly, the lack of diversity in our sample is another limitation. Most participants were of Finnish origin and identified as female, and previous research suggests potential gender differences in forming attachments to pets^62–65^. Women are also consistently reported to be at a higher risk for affective disorders^66^. However, the traditional measures of affective disorders may be more sensitive to women’s expression of symptoms and thus, men’s distress may be under-reported^67,68^. Nevertheless, future studies need to include more diverse samples to examine the generalizability of our findings to other cultures and genders. Thirdly, it is worth noting that the analyses relied on self-reported online questionnaires, and participation in the study required effort. Consequently, the data may be subject to bias, favoring more enthusiastic and engaged pet owners. Participants were recruited exclusively through social media, potentially excluding individuals not using these platforms. Fourthly, the item pool of the scale was formulated and selected qualitatively based on descriptions from Finnish dog owners regarding their experiences during the puppy stage. Therefore, it is possible that certain aspects of the phenomenon, such as those related to other cultures, may not have been fully covered.

In conclusion, the puppy blues scale was reliable and valid, with good internal consistency and test-retest reliability. The scale conceptually distinguishes three dimensions: Frustration, Anxiety, and Weariness. The three factors identified accounted for a large proportion of the variance in puppy blues and demonstrated good internal consistency and test-retest reliability. The scale also demonstrated good predictive validity, as all factors showed linear associations with the subjective burden score. The scale’s convergent validity was supported by the observed associations between factor scores and meaningful psychological constructs in hypothesis testing. Overall, the findings support the reliability, validity, and utility of the self-report scale for measuring the targeted construct. The scale can be a valuable tool for researchers and practitioners in the field of human-companion animal bonds and mental health. Future studies could explore the prevalence and predisposing factors of the puppy blues, explore the scale’s generalizability to other cultures and genders, and establish the concurrent validity of the scale in relation to other measures. Overall, the development and validation of the puppy blues scale is an important step towards understanding the experiences of puppy owners and providing support for those who may be struggling with distressing dysphoric symptoms amid significant life changes. While much remains to be explored, our findings here demonstrate that parallels exist in experiences related to the postpartum period for parents and the caretakers of puppies.

### Supplementary Information

Supplementary Information

## Data Availability

The datasets used and analyzed during the current study are available from the corresponding author on reasonable request.

## Code availability

The underlying code for this study is available in this paper’s supplementary methods. Any additional information required to reanalyze the data reported in this paper is available from the corresponding author upon request.

## References

[1] Zilcha-Mano S, Mikulincer M, Shaver PR (2012). Pets as safe havens and secure bases: the moderating role of pet attachment orientations. J. Res. Pers..

[2] Cavanaugh LA, Leonard HA, Scammon DL (2008). A tail of two personalities: how canine companions shape relationships and well-being. J. Bus. Res..

[3] Coy AE, Green JD (2018). Treating pets well: the role of attachment anxiety and avoidance. Hum. Anim. Interact. Bull..

[4] Coy AE, Green JD, Behler AMC (2021). Why can’t i resist those “puppy dog” (or “kitty cat”) eyes? A study of owner attachment and factors associated with pet obesity. Animals.

[5] Schöberl I (2012). Effects of owner-dog relationship and owner personality on cortisol modulation in human-dog dyads. Anthrozoos.

[6] Britton K (2018). Caregiving for a companion animal compared to a family member: burden and positive experiences in caregivers. Front. Vet. Sci..

[7] Christiansen SB, Kristensen AT, Sandøe P, Lassen J (2013). Looking after chronically III dogs: impacts on the Caregiver’s life. Anthrozoos.

[8] Spitznagel MB, Jacobson DM, Cox MD, Carlson MD (2017). Caregiver burden in owners of a sick companion animal: a cross-sectional observational study. Vet. Rec..

[9] Costa AG, Nielsen T, Christley R, Hazel S (2023). The good, the bad, the helpful: qualitative exploration of the australian puppy-raising experience through longitudinal surveys. Anthrozoos.

[10] Furtado, T., Casey, R., Upjohn, M. & Christley, R. In the doghouse? an exploration of online discussions around the challenges of human-dog relationships. *Soc. Anim.*10.1163/15685306-bja10153 (2023).

[11] McConnell AR, Paige Lloyd E, Humphrey BT (2019). We are family: viewing pets as family members improves wellbeing. Anthrozoos.

[12] Salonen M (2023). Breed, age, and social environment are associated with personality traits in dogs. iScience.

[13] Tosto V (2023). Maternity blues: a narrative review. J. Pers. Med..

[14] O’Hara MW, Wisner KL (2014). Perinatal mental illness: definition, description and aetiology. Best. Pr. Res. Clin. Obstet. Gynaecol..

[15] Zilcha-Mano S, Mikulincer M, Shaver PR (2011). An attachment perspective on human-pet relationships: conceptualization and assessment of pet attachment orientations. J. Res. Pers..

[16] Topál J, Miklósi Á, Csányi V, Dóka A (1998). Attachment behavior in dogs (Canis familiaris): a new application of Ainsworth’s (1969) Strange Situation Test. J. Comp. Psychol..

[17] Molebatsi K, Ndetei DM, Opondo PR (2017). Caregiver burden and correlates among caregivers of children and adolescents with psychiatric morbidity: a descriptive cross sectional study. J. Child Adolesc. Ment. Health.

[18] Liu M, Lambert CE, Lambert VA (2007). Caregiver burden and coping patterns of Chinese parents of a child with a mental illness. Int. J. Ment. Health Nurs..

[19] Buller K, Ballantyne KC (2020). Living with and loving a pet with behavioral problems: Pet owners’ experiences. J. Vet. Behav..

[20] Barrios CL, Gornall V, Bustos-López C, Cirac R, Calvo P (2022). Creation and validation of a tool for evaluating caregiver burnout syndrome in owners of dogs (Canis lupus familiaris) diagnosed with behavior disorders. Animals.

[21] Puyané M (2022). Personality traits as a risk factor for postpartum depression: a systematic review and meta-analysis. J. Affect Disord..

[22] Bradley R, Slade P (2011). A review of mental health problems in fathers following the birth of a child. J. Reprod. Infant Psychol..

[23] Halbreich U (2005). The association between pregnancy processes, preterm delivery, low birth weight, and postpartum depressions—the need for interdisciplinary integration. Am. J. Obstet. Gynecol..

[24] Dietz L, Arnold A-MK, Goerlich-Jansson VC, Vinke CM (2018). The importance of early life experiences for the development of behavioural disorders in domestic dogs. Behaviour.

[25] Cox JL, Holden JM, Sagovsky R (1987). Detection of postnatal depression. Br. J. Psychiatry.

[26] Cockshaw WD, Thorpe KJ, Giannotti M, Hazell-Raine K (2023). Factor structure of the Edinburgh Postnatal Depression Scale in a large population-based sample of fathers. J. Affect Disord..

[27] Salonen M (2021). Reliability and validity of a dog personality and unwanted behavior survey. Animals.

[28] Ståhl A (2023). Pet and owner personality and mental wellbeing associate with attachment to cats and dogs. iScience.

[29] Harris PA (2009). Research electronic data capture (REDCap)—a metadata-driven methodology and workflow process for providing translational research informatics support. J. Biomed. Inf..

[30] Harris PA (2019). The REDCap consortium: Building an international community of software platform partners. J. Biomed. Inf..

[31] Wright, K. Corrgram: Plot a Correlogram. https://cran.r-project.org/web/packages/corrgram/index.html (2021).

[32] Bliese, P., Chen, G., Downes, P., Schepker, D. & Lang, J. Package multilevel: multilevel functions. https://cran.r-project.org/web/packages/multilevel/index.html (2022).

[33] Revelle, W. R. Psych: procedures for personality and psychological research. https://cran.r-project.org/package=psych (2023).

[34] Bernaards CA, Jennrich RI (2005). Gradient projection algorithms and software for arbitrary rotation criteria in factor analysis. Educ. Psychol. Meas..

[35] Rosseel, Y. Lavaan: latent variable analysis. https://cran.r-project.org/web/packages/lavaan/index.html (2023).

[36] Shrout PE, Fleiss JL (1979). Intraclass correlations: uses in assessing rater reliability. Psychol. Bull..

[37] Koo TK, Li MY (2016). A guideline of selecting and reporting intraclass correlation coefficients for reliability research. J. Chiropr. Med..

[38] Fox, J. & Dusa, A. Polycor: polychoric and polyserial correlations. https://cran.r-project.org/package=polycor (2022).

[39] Wickham, H. et al. Ggplot2: create elegant data visualisations using the grammar of graphics. https://cran.r-project.org/web/packages/ggplot2/index.html (2024).

[40] Kassambara, A. Package ggpubr: ‘ggplot2’ based publication ready plots. https://cran.r-project.org/web/packages/ggpubr/index.html (2023).

[41] Kuhn, M. et al. Caret: classification and regression training. https://cran.r-project.org/web/packages/caret/index.html (2023).

[42] Walker WR, Vogl RJ, Thompson CP (1997). Autobiographical memory: unpleasantness fades faster than pleasantness over time. Appl. Cogn. Psychol..

[43] Walker WR, Skowronski JJ, Thompson CP (2003). Life is pleasant—and memory helps to keep it that way!. Rev. Gen. Psychol..

[44] Smorti M, Ponti L, Pancetti F (2019). A comprehensive analysis of post-partum depression risk factors: the role of socio-demographic, individual, relational, and delivery characteristics. Front. Public Health.

[45] Norhayati MN, Hazlina Nik, Asrenee NH, Wan AR, Emilin WMA (2015). Magnitude and risk factors for postpartum symptoms: a literature review. J. Affect Disord..

[46] Konstabel K, Lönnqvist JE, Walkowitz G, Konstabel K, Verkasalo M (2012). The ‘Short Five’ (S5): measuring personality traits using comprehensive single items. Eur. J. Pers..

[47] Spitzer RL, Kroenke K, Williams JBW, Löwe B (2006). A brief measure for assessing generalized anxiety disorder. Arch. Intern Med.

[48] Kohout FJ, Berkman LF, Evans DA, Cornoni-Huntley J (1993). Two shorter forms of the CES-D Depression Symptoms Index. J. Aging Health.

[49] R Core Team. *R: A Language and Environment for Statistical Computing* (R Foundation for Statistical Computing, 2022).

[50] Wickham, H., François, R., Henry, L., Müller, K. & Vaughan, D. Dplyr: a grammar of data manipulation. https://cran.r-project.org/web/packages/dplyr/index.html (2023).

[51] Gray MJ, Litz BT, Hsu JL, Lombardo TW (2004). Psychometric properties of the life events checklist. Assessment.

[52] Rezaie-Keikhaie K (2020). Systematic review and meta-analysis of the prevalence of the maternity blues in the postpartum period. J. Obstet. Gynecol. Neonatal Nurs..

[53] Liu X, Wang S, Wang G (2022). Prevalence and risk factors of postpartum depression in women: a systematic review and meta‐analysis. J. Clin. Nurs..

[54] Ruohomäki A (2018). The association between gestational diabetes mellitus and postpartum depressive symptomatology: a prospective cohort study. J. Affect Disord..

[55] Oliva JL, Johnston KL (2021). Puppy love in the time of Corona: Dog ownership protects against loneliness for those living alone during the COVID-19 lockdown. Int. J. Soc. Psychiatry.

[56] Powell L (2019). Companion dog acquisition and mental well-being: a community-based three-arm controlled study. BMC Public Health.

[57] Duvall Antonacopoulos NM (2017). A longitudinal study of the relation between acquiring a dog and loneliness. Soc. Anim..

[58] Brannan AM, Heflinger CA, Bickman L (1997). The caregiver strain questionnaire: measuring the impact on the family of living with a child with serious emotional disturbance. J. Emot. Behav. Disord..

[59] Cicchetti DV (1994). Guidelines, criteria, and rules of thumb for evaluating normed and standardized assessment instruments in psychology. Psychol. Assess..

[60] López FA (2021). Effect of delayed-release and extended-release methylphenidate on caregiver strain and validation of psychometric properties of the caregiver strain questionnaire: results from a phase 3 trial in children with attention-deficit/hyperactivity disorder. J. Child Adolesc. Psychopharmacol..

[61] Yang R (2021). Psychometric properties of the caregiver strain questionnaire among chinese parents of children with ADHD or ASD. Gen. Psychiatr..

[62] Reevy GM, Delgado MM (2015). Are emotionally attached companion animal caregivers conscientious and neurotic? factors that affect the human–companion animal relationship. J. Appl. Anim. Welf. Sci..

[63] Reevy GM, Delgado MM (2020). The relationship between neuroticism facets, conscientiousness, and human attachment to pet cats. Anthrozoos.

[64] Dotson MJ, Hyatt EM (2008). Understanding dog-human companionship. J. Bus. Res..

[65] Smolkovic I, Fajfar M, Mlinaric V (2012). Attachment to pets and interpersonal relationships: Can a four-legged friend replace a two-legged one?. J. Eur. Psychol. Stud..

[66] Faravelli C, Alessandra Scarpato M, Castellini G, Lo Sauro C (2013). Gender differences in depression and anxiety: the role of age. Psychiatry Res..

[67] Brownhill S, Wilhelm K, Barclay L, Schmied V (2005). Big build’: hidden depression in men. Aust. N. Z. J. Psychiatry.

[68] Fisher SD (2021). Expanding the international conversation with fathers’ mental health: toward an era of inclusion in perinatal research and practice. Arch. Women’s Ment. Health.

