# Supplementary material for: Development and validation of the puppy blues scale measuring temporary affective disturbance resembling baby blues
Source: Npj Ment Health Res. 2024 Jun 7;3:27. doi: 10.1038/s44184-024-00072-z (PMC11161525; doi:10.1038/s44184-024-00072-z)
Supplement: Supplementary file 1 — Supplementary Information [file 44184_2024_72_MOESM1_ESM.pdf]

Supplementary Table 1. Inter-item correlations.

	1	2	3	4	5	6	7	8	9	10	11	12	13	14	15
1. Difficulty in taking care of the puppy	1.00	.35	.55	.63	.36	.40	.40	.64	.26	.44	.18	.52	.48	.59	.45
2. Puppy well-being worries		1.00	.21	.41	.14	.39	.19	.34	.27	.23	.48	.39	.43	.30	.20
3. Irritation towards the puppy			1.00	.53	.39	.36	.43	.50	.17	.32	.11	.45	.38	.47	.43
4. Feeling of inadequacy				1.00	.31	.62	.38	.54	.20	.34	.24	.69	.65	.50	.39
5. Considering giving away the puppy					1.00	.22	.30	.34	.15	.26	.07	.28	.23	.41	.56
6. Fear of "ruining" the puppy						1.00	.34	.41	.24	.28	.30	.61	.69	.41	.30
7. Challenges in forming an emotional bond							1.00	.38	.18	.25	.08	.36	.32	.41	.39
8. Exhaustion								1.00	.41	.48	.22	.50	.46	.58	.44
9. Sleep problem									1.00	.33	.21	.24	.26	.31	.19
10. Anxiety about required attention and time										1.00	.16	.35	.33	.44	.39
11. Wary											1.00	.31	.39	.21	.08
12. Guilt as a dog owner												1.00	.67	.52	.40
13. Worry about raising puppy correctly													1.00	.50	.33
14. Cumbersome														1.00	.51
15. Regret															1.00

Supplementary Table 2. Item-total correlations.

Item (Abbreviated)	Item total correlation	Cronbach's Alpha without the item	N
1. Difficulty in taking care of the puppy	.70	.88	1778
2. Puppy well-being worries	.50	.89	1778
3. Irritation towards the puppy	.58	.89	1778
4. Feeling of inadequacy	.74	.88	1778
5. Considering giving away the puppy	.41	.89	1778
6. Fear of "ruining" the puppy	.65	.88	1778
7. Challenges in forming an emotional bond	.48	.89	1778
8. Exhaustion	.68	.88	1778
9. Sleep problem	.37	.89	1778
10. Anxiety about required attention and time	.49	.89	1778
11. Wary	.34	.90	1778
12. Guilt as a dog owner	.73	.88	1778
13. Worry about raising puppy correctly	.72	.88	1778
14. Cumbersome	.68	.88	1778
15. Regret	.53	.89	1778

Supplementary Table 3. Test-retest reliabilities in the puppy blues questionnaire.

Item (Abbreviated)	Test-retest reliability	
	**Pearson correlation**	**Intraclass correlation**
1. Difficulty in taking care of a puppy	.71	.71
2. Puppy well-being worries	.67	.67
3. Irritation towards the puppy	.69	.69
4. Feeling of inadequacy	.71	.70
5. Considering giving away the puppy	.84	.84
6. Fear of "ruining" the puppy	.65	.65
7. Challenges in forming an emotional bond	.65	.66
8. Exhaustion	.72	.72
9. Sleep problem	.44	.44
10. Anxiety about required attention and time	.67	.67
12. Guilt as a dog owner	.68	.67
13. Worry about raising puppy correctly	.66	.66
14. Cumbersome	.66	.65
15. Regret	.70	.70
REMOVED ITEMS		
11. Wary	.59	.59

Supplementary Figure 1. Boxplots of the association between factor scores and subjective burden. Association of A) anxiety, B) frustration, C) weariness with the owner's subjective burden from 1 = not at all to 7 = extremely.

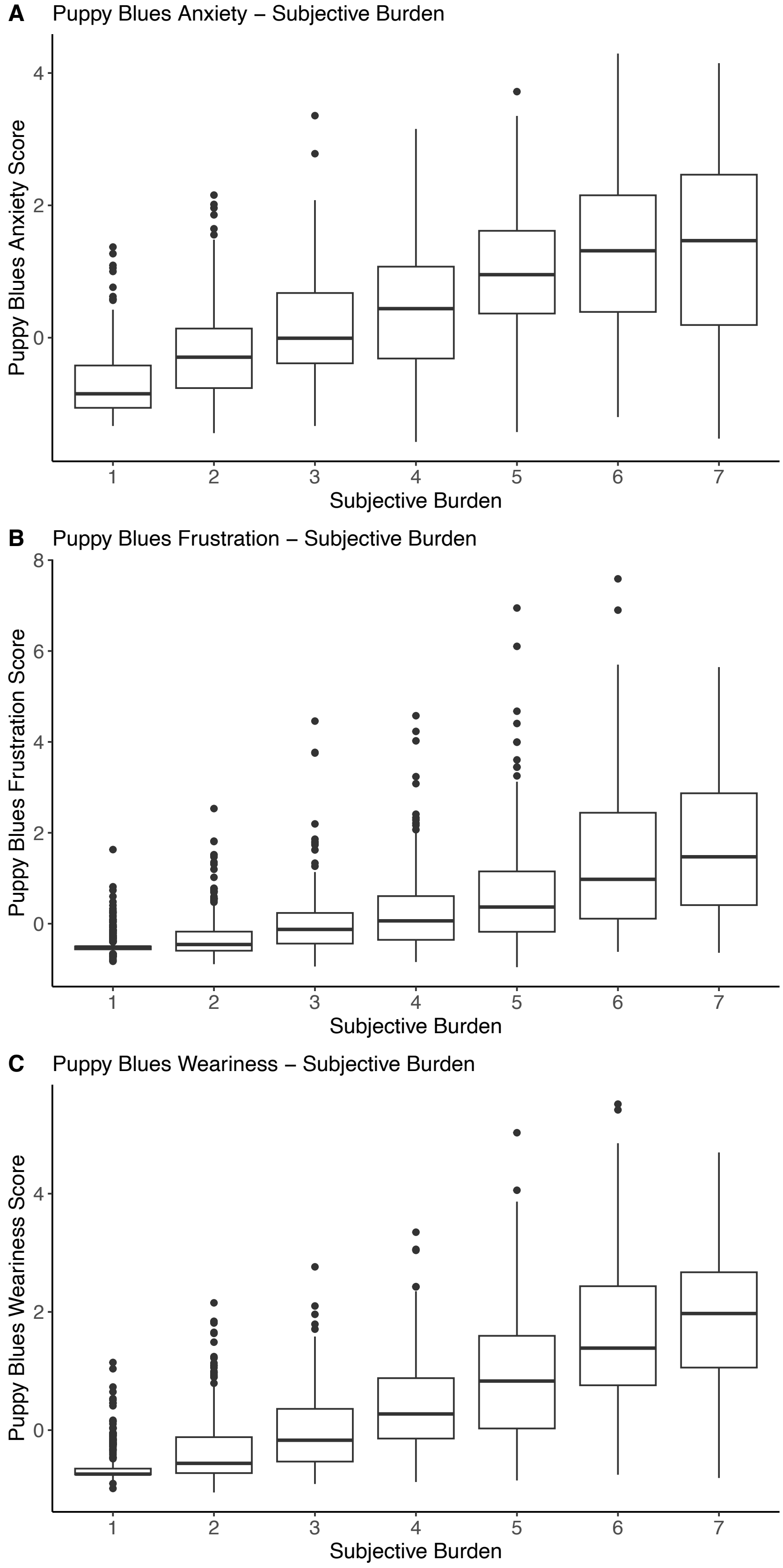

Supplementary Table 4. The factor loadings in cross-validation analysis on test sample.

Item no*	Item (Abbreviated)	Anxiety	Frustration	Weariness
6	Fear of "ruining" the puppy	**.92**	-.04	-.07
13	Worry about raising puppy correctly	**.92**	-.09	.05
12	Guilt as a dog owner	**.75**	.16	.02
4	Feeling of inadequacy	**.68**	.24	.03
2	Concern for the puppy's well-being	**.46**	-.12	.24
5	Considering giving away the puppy	-.01	**.88**	.00
15	Regret	.03	**.82**	.06
3	Irritation towards the puppy	.15	**.61**	.10
7	Challenges in forming an emotional bond	.19	**.48**	.07
1	Difficulty in taking care of a puppy	.16	**.39**	**.41**
14	Cumbersome	.28	**.34**	**.39**
8	Exhaustion	-.01	.19	**.79**
9	Sleep problem	.03	-.20	**.79**
10	Anxiety about required attention and time	.08	.20	**.58**

Loadings >.30 are in bold.

*Item order in the questionnaire

Supplementary Table 5. Confirmatory factor analysis (CFA) fit indices.

Model	CFI	TLI	RMSEA	SRMR
3-factor, item wary removed[1]	.922	.903	.082	.050
1-factor[2]	.783	.746	.149	.091
2-factor[2]	.893	.872	.106	.068
3-factor[2]	.902	.877	.104	.064
4-factor[2]	.930	.911	.089	.058
bifactorial, 3 subfactors[2]*	.941	.913	.088	.048
3-factor revised (all items with <.30 loadings removed)[2]	.909	.882	.111	.068
1-factor item wary removed[2]	.814	.780	.145	.078
2-factor item wary removed[2]	.917	.899	.098	.055
3-factor item wary removed[2]	.924	.902	.097	.053
4-factor item wary removed[2]	.784	.735	.159	.354
bifactorial, 3 subfactors, item wary removed[2]*	.965	.948	.070	.0.37

[1] Cross-validation (performed to a sample when data was split into two samples)

[2] Test of dimensionality (performed to the separate data of 326 respondents)

*a Heywood case

Supplementary Table 6. Distribution of differences in factor scores in responses compared to the current moment (dog is 1–2-year old) against responses recalling the puppy period (n=326). SD= Standard deviation.

Factor	< -1 SD	-1 to -0.5 SD	-0.5 to 0 SD	0 to 0.5 SD	0.5 to 1 SD	> 1 SD	NA
Anxiety	54	36	151	67	10	2	6
Frustration	71	56	130	53	9	1	6
Weariness	66	50	134	55	13	2	6

Supplementary Figure 2. Scatter plots of factor scores against the amount of time that had passed since the birth of the dog. A) frustration, B) anxiety, C) weariness plotted against the time from birth in years.

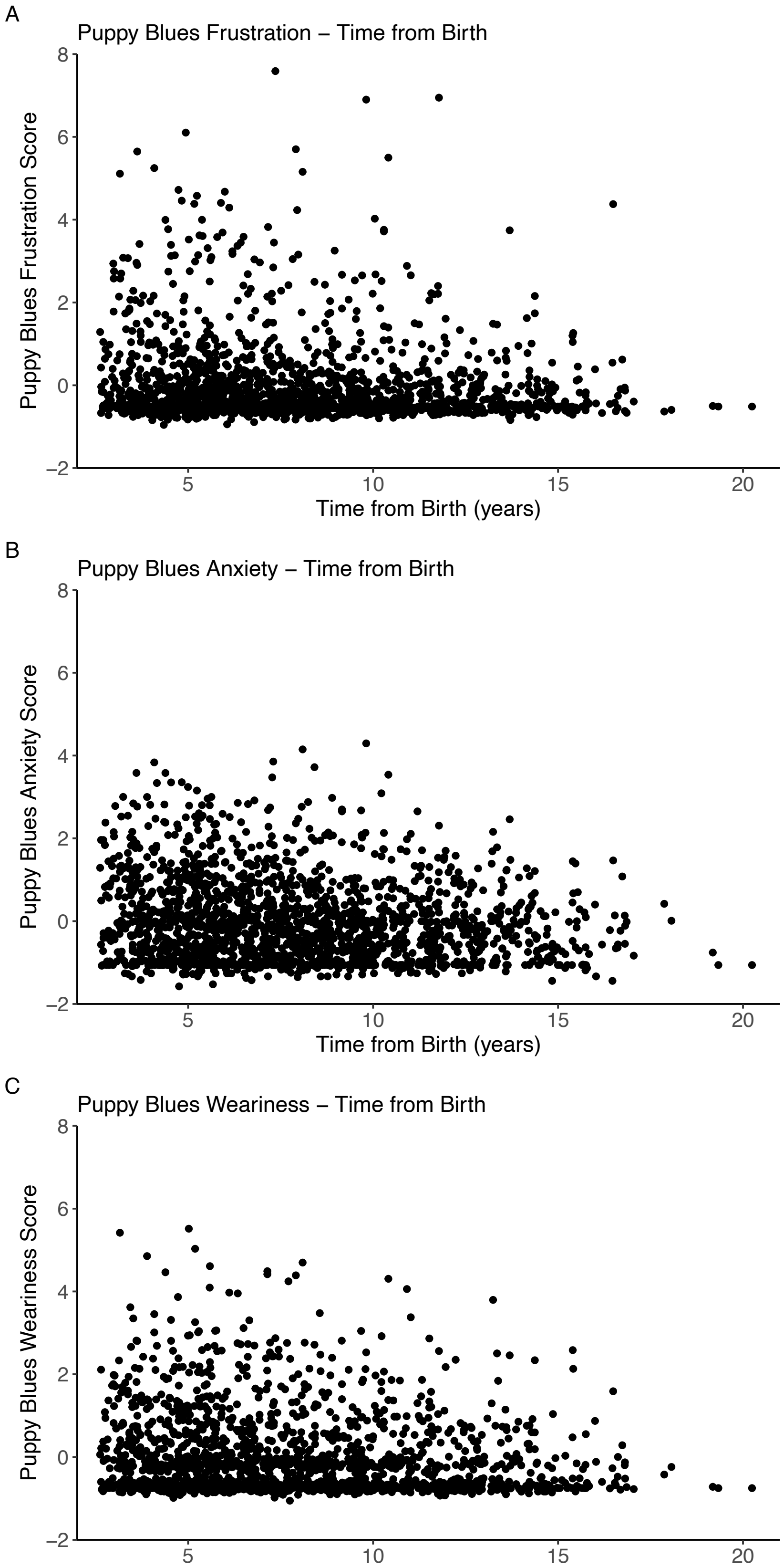

Supplementary Notes

Questionnaire

The following questions ask about your feelings and thoughts **during your dog's puppyhood**. For each statement, you will be asked to indicate how often you felt or thought a certain way when your dog was a puppy. Think of the time when you felt the most distressed.

Data protection

Hereby, I accept that my personal information, the information of my dog and all the data collected with this questionnaire I have provided is transferred to Canine Genetics research group at the University of Helsinki and used in scientific research. Read the privacy policy of the Canine Genetics Group (only in Finnish).

During your dog's puppyhood, how often…	Almost never or never	Some-times	Fairly often	Almost all the time	I don't know
1. …did the puppy and taking care of the puppy feel more difficult than you had expected?	0	1	2	3	-
2. … were you worried about the well-being of the puppy?	0	1	2	3	-
3. …did you feel irritation/anger towards the puppy?	0	1	2	3	-
4. …did you feel inadequate and incompetent as a dog owner?	0	1	2	3	-
5. …did you think about giving away the puppy?	0	1	2	3	-
6. …did you worry that you would "ruin" the puppy with your actions?	0	1	2	3	-
7. …did building an emotional bond and connection with the puppy seem difficult?	0	1	2	3	-
8. …did you feel exhausted?	0	1	2	3	-
9. …did you have trouble sleeping, even when the puppy slept well?	0	1	2	3	-
10. … were you anxious that the puppy was taking up all your attention and time?	0	1	2	3	-
11. …did you watch over the puppy in case something bad would happen to the puppy?	0	1	2	3	-
12. …did you feel bad conscience and guilt towards yourself as a dog owner when things didn't go as you expected?	0	1	2	3	-
13. …did you worry about caring for and raising the puppy "correctly"?	0	1	2	3	-
14. …did everything you do feel like an effort?	0	1	2	3	-
15. …did you question or regret your choice to get a puppy?	0	1	2	3	-

16. Overall, when you think about the time when you felt the most distressed during your dog's puppyhood, how burdened did you feel?

Not at all Moderately Extremely

1	2	3	4	5	6	7

17. For how long did the above-mentioned feelings related to puppyhood continue equally strong?

I didn't have significantly negative feelings during the puppyhood

0 to 4 weeks

1 to 5 months

6 months - a year

over a year

18. Which of the following did you do **to ease the negative feelings** associated with puppyhood:

I didn't have significantly negative feelings during the puppyhood

I didn't feel like anything helped

I talked to my friends or family members about my feelings

I took time off from the puppy

I tried to see my feelings as natural and reasonable

I attended to dog training classes

I talked to the breeder about my feelings

I tried to accept my feelings

I tried to learn to live with my feelings

I received dog care help from others

I searched information

I expressed my negative feelings

I tried to distract myself by doing other things

I tried to see things in a more positive light

I talked to other dog owners about my feelings

I waited for the feelings to pass by themselves

Something else, what? _________

Did something else significant happen in your life during your dog's puppyhood? There are various stressful things that sometimes happen to people listed below. For each event, indicate to what extent you felt the event had a positive or negative impact on your life when your dog was a puppy.

–3 = very negative impact

0 = did not happen or the event had no positive or negative impact

+3 = very positive impact

19. A natural disaster
20. A pandemic
21. Threat of war
22. Pregnancy or birth of a child
23. Significantly changed work situation
24. Significantly changed financial situation
25. A significant change in a close relationship
26. Own illness or injury
27. Illness or injury of a close family member or a friend
28. Death of a close family member or a friend
29. Some other very stressful event or experience

30. How long did you have to wait for the puppy? (Referring to a time from the first contact with the breeder/breeders or other place of obtainment to the arrival of the puppy)

    Less than 4 months
    4-6 months
    6 months to 1 year
    1–1.5 years
    1.5–2 years
    2–3 years
    More than 3 years

Supplementary Methods

R code

```
#### PACKAGES ####

library(dplyr) #for data handling
library(psych) #for conducting corrected item-total correlation, exploratory fa
ctor analysis, reliability analysis and correlations
library(GPArotation) #for choosing the rotation method "oblimin" in EFA
library(corrgram) #for visualizing inter-item correlations
library(multilevel) #for calculating item-total correlations
library(polycor) # for calculating polyserial correlations
library(ggpubr) #for data visualization
library(caret) #for splitting the data
library(lavaan) #for conducting confirmatory factor analysis
library(ggplot2) #for data visualization

#### DATA EDITS ####

#First read in data
puppyblues <- read_table()

#Select puppy blues survey items
puppyblues.col <- puppyblues[,c(13:27)]

####ITEM REDUCTION ANALYSIS####

####Inter-item correlations####
inter_item_cor <- cor(puppyblues.col[,c(1:15)],use="pairwise.complete.obs")
#Visualization of the inter-item correlations
corrgram(puppyblues.col, order=TRUE,
         text.panel=panel.txt,
         lower.panel= panel.shade, upper.panel=corrgram::panel.cor)

####Item-total correlation####
item_total_cor <- item.total(puppyblues.col)

#Corrected item-total correlation
psych::alpha(puppyblues.col)

###### DATA SUITABILITY FOR FA AND NUMBER OF FACTORS TO EXTRACT #######

###Is the data factorable?
KMO(puppyblues.col)
#Yes! overall MSA = .92 (marvelous)
#All over .80, ranging from .81 to .96 (meritorious to marvelous)

###Scree test to determine the number of factors
scree(puppyblues.col,factors=T,pc=F)
#Scree suggests 4 factors

###MAP to indicate the number of factors
```

```
VSS(puppyblues.col, n=15, fm="ml", n.obs=1799, title="number of factors", cor="
poly")
#MAP reaches minimum with 3 factors

####### FACTOR ANALYSIS #########

#Comparison of factor structures via Goldberg's hierarchical tree and
#Comparison of fit indices between structures

#Run all possible factor structures from one factor to more factors
#(at least n+1) than suggested by scree and MAP)

#One factor with mean maximum likelihood estimation and mean imputation
puppyblues_1 <- fa(puppyblues.col, nfactors=1,scores="Thurstone", warnings=TRUE
, fm="ml", cor="poly", missing=T, impute="mean")

#Show results of EFA
print(puppyblues_1, cut=0.3)

#Similarly done with 2-5 factors
puppyblues_2 <- fa(puppyblues.col, nfactors=2,scores="Thurstone", warnings=TRUE
, fm="ml", cor="poly", missing=T, impute="mean")
puppyblues_3 <- fa(puppyblues.col, nfactors=3,scores="Thurstone", warnings=TRUE
, fm="ml", cor="poly", missing=T, impute="mean")
puppyblues_4 <- fa(puppyblues.col, nfactors=4,scores="Thurstone", warnings=TRUE
, fm="ml", cor="poly", missing=T, impute="mean")
puppyblues_5 <- fa(puppyblues.col, nfactors=5,scores="Thurstone", warnings=TRUE
, fm="ml", cor="poly", missing=T, impute="mean")

print(puppyblues_2, cut=0.3)
print(puppyblues_3, cut=0.3)
print(puppyblues_4, cut=0.3)
print(puppyblues_5, cut=0.3)

#-> Three factor solution selected

###### Cronbach's alpha & Guttman's lambda #######

#Assigns items to factors
keys.list <- list(frustration=c("burden_care_difficult", "burden_irritation_ang
er", "burden_inadequacy",
                                "burden_relinquishment", "burden_connect_diffic
ult", "burden_exhausted",
                                "burden_cumbersome", "burden_regret", "-burden_
wary"),
                  anxiety=c("burden_concerned_wellbeing", "burden_inadequacy",
"burden_fear_ruin",
                            "burden_wary", "burden_guilt", "burden_raise_right"
),
                  weariness= c("burden_care_difficult", "burden_sleep_problem",
"burden_anxious_attention", "burden_exhausted"))

#Calculate Cronbach's alpha and Guttman's lambda for the factors
scores <- scoreItems(keys.list,puppyblues.col,missing=T, impute="mean",min=1,ma
```

```
x=3)
print(scores,short=T)

#-> Based on cross-loadings and item-total correlations item burden_wary is
removed.
#The item burden_wary did not load uniquely on individual factors but loaded on
to all factors.

#### REVISED FACTOR ANALYSIS ####

#Select survey items with the exclusion of the item burden_wary
puppyblues.col.revised <- puppyblues[,c(13:22,24:27)]

#All steps of analysis done as previously

#Is the data factorable?
KMO(puppyblues.col.revised)
#Yes! overall MSA = .93 (marvelous)
#All over .80, ranging from .87 to .96 (meritorious to marvelous)

###Scree test to determine the number of factors
scree(puppyblues.col.revised,factors=T,pc=F)
#Scree suggests 4 factors
VSS(puppyblues.col.revised, n=14, fm="ml", n.obs=1799, title="number of factors
", cor="poly")
#MAP reaches minimum with 3 factors

puppyblues_1_revised <- fa(puppyblues.col.revised, nfactors=1,scores="Thurstone
", warnings=TRUE, fm="ml", cor="poly", missing=T, impute="mean")
puppyblues_2_revised <- fa(puppyblues.col.revised, nfactors=2,scores="Thurstone
", warnings=TRUE, fm="ml", cor="poly", missing=T, impute="mean")
puppyblues_3_revised <- fa(puppyblues.col.revised, nfactors=3,scores="Thurstone
", warnings=TRUE, fm="ml", cor="poly", missing=T, impute="mean")
puppyblues_4_revised <- fa(puppyblues.col.revised, nfactors=4,scores="Thurstone
", warnings=TRUE, fm="ml", cor="poly", missing=T, impute="mean")
puppyblues_5_revised <- fa(puppyblues.col.revised, nfactors=5,scores="Thurstone
", warnings=TRUE, fm="ml", cor="poly", missing=T, impute="mean")

print(puppyblues_1_revised, cut=0.3)
print(puppyblues_2_revised, cut=0.3)
print(puppyblues_3_revised, cut=0.3)
print(puppyblues_4_revised, cut=0.3)
print(puppyblues_5_revised, cut=0.3)

###### Cronbach's alpha & Guttman's lambda for revised EFA #######

#Assigns items to three factors
keys.list2 <- list(frustration=c("burden_care_difficult", "burden_irritation_an
ger",
                                 "burden_relinquishment", "burden_connect_diffi
cult",
                                 "burden_cumbersome", "burden_regret"),
                   anxiety=c("burden_concerned_wellbeing", "burden_inadequacy",
"burden_fear_ruin",
```

```
                              "burden_guilt", "burden_raise_right"),
                    weariness= c("burden_care_difficult", "burden_cumbersome", "
burden_sleep_problem",
                                 "burden_anxious_attention", "burden_exhausted")
)

scores.revised <- scoreItems(keys.list2,puppyblues.col.revised,missing=T, imput
e="mean",min=1,max=3)
# Cronbach's alpha and Guttman's lambda (+ some others) for all 3 factors
print(scores.revised,short=T)

###### Factor scores for revised EFA ########
#Calculate factor scores for individuals
puppyblues.scores.revised <- factor.scores(puppyblues.col.revised, puppyblues_3
_revised, method="tenBerge", impute="mean")

#Merge scores with the original data that also includes at least individuals' u
nique ids
puppyblues.scores.revised.2 <- cbind(puppyblues, puppyblues.scores.revised$scor
es)
summary(puppyblues.scores.revised.2)

#Rename factors
names(puppyblues.scores.revised.2)[names(puppyblues.scores.revised.2) == "1"] <
- "puppyblues_anxiety"
names(puppyblues.scores.revised.2)[names(puppyblues.scores.revised.2) == "2"] <
- "puppyblues_frustration"
names(puppyblues.scores.revised.2)[names(puppyblues.scores.revised.2) == "3"] <
- "puppyblues_weariness"

#### TEST OF DIMENSIONALITY: CFA FOR A SEPARATE DATA ####
#Read in the data
puppy_adult <- read_table ()

#Select puppy blues survey items
puppyhood.col <- puppy_adult[,c(6:20)]
#Select puppy blues survey items without burden_wary for the revised models
puppyhood.col.revised <- puppy_adult[,c(6:15,17:20)]

#CFA with the three factor revised model (burden_wary removed)
threefactormodel_revised <- 'frustration =~ burden_care_difficult + burden_irri
tation_anger + burden_inadequacy + burden_relinquishment
                  + burden_connect_difficult + burden_exhausted + burden_cumber
some + burden_regret
  anxiety =~ burden_concerned_wellbeing + burden_inadequacy + burden_fear_ruin
+ burden_guilt + burden_raise_right
  weariness =~ burden_care_difficult + burden_sleep_problem + burden_anxious_at
tention + burden_exhausted'
threefactors_revised <- cfa(threefactormodel_revised, data=puppyhood.col.revise
d,std.lv=TRUE)
summary(threefactors_revised,fit.measures=TRUE,standardized=TRUE)
parameterEstimates(threefactors_revised, standardized = TRUE)

# CFA for the competing original three factor model
```

```
model_3factors <- 'frustration =~ burden_care_difficult + burden_irritation_ang
er + burden_inadequacy + burden_relinquishment
                  + burden_connect_difficult + burden_exhausted + burden_cumber
some + burden_regret + -burden_wary
  anxiety =~ burden_concerned_wellbeing + burden_inadequacy + burden_fear_ruin
+ burden_wary + burden_guilt + burden_raise_right
  weariness =~ burden_care_difficult + burden_sleep_problem + burden_anxious_at
tention + burden_exhausted'
threefactors <- cfa(model_3factors, data=puppyhood.col,std.lv=TRUE)
summary(threefactors,fit.measures=TRUE,standardized=TRUE)
parameterEstimates(threefactors, standardized = TRUE)

# CFA with a competing four-factor model
model_4factors <- 'anxiety =~ burden_concerned_wellbeing + burden_inadequacy +
burden_fear_ruin + burden_wary + burden_guilt + burden_raise_right
          frustration =~ burden_relinquishment + burden_connect_difficult + bur
den_regret
    weariness =~ burden_care_difficult + burden_exhausted + burden_sleep_proble
m + burden_anxious_attention + burden_cumbersome
overwhelm =~ burden_care_difficult + burden_irritation_anger + burden_inadequac
y'
fourfactors <- cfa(model_4factors, data=puppyhood.col,std.lv=TRUE)
summary(fourfactors,fit.measures=TRUE,standardized=TRUE)
parameterEstimates(fourfactors, standardized = TRUE)

# CFA with a competing two factor model
model_2factors <- 'frustration =~ burden_care_difficult + burden_irritation_ang
er + burden_inadequacy + burden_relinquishment
                  + burden_connect_difficult + burden_exhausted + burden_sleep_
problem + burden_anxious_attention
                  + burden_cumbersome + burden_regret
                  anxiety =~ burden_concerned_wellbeing + burden_inadequacy + b
urden_fear_ruin + burden_wary
                  + burden_guilt + burden_raise_right'
twofactors <- cfa(model_2factors, data=puppyhood.col,std.lv=TRUE)
summary(twofactors,fit.measures=TRUE,standardized=TRUE)
parameterEstimates(twofactors, standardized = TRUE)

# CFA with a competing one factor model
model_1factor <- 'puppyblues =~ burden_care_difficult + burden_concerned_wellbe
ing + burden_irritation_anger + burden_inadequacy
              + burden_relinquishment + burden_fear_ruin + burden_connect_diffi
cult + burden_exhausted
              + burden_sleep_problem + burden_anxious_attention + burden_wary +
burden_guilt + burden_raise_right
              + burden_cumbersome + burden_regret'
onefactor <- cfa(model_1factor, data=puppyhood.col,std.lv=TRUE)
summary(onefactor,fit.measures=TRUE,standardized=TRUE)
parameterEstimates(onefactor, standardized = TRUE)

# CFA with a competing higher-order factor structure (bifactor model)
model_bifactor <- 'frustration =~ burden_care_difficult + burden_irritation_ang
er + burden_inadequacy + burden_relinquishment
                  + burden_connect_difficult + burden_exhausted + burden_cumber
```

```
some + burden_regret + -burden_wary
                  anxiety =~ burden_concerned_wellbeing + burden_inadequacy + b
urden_fear_ruin + burden_wary + burden_guilt + burden_raise_right
                  weariness =~ burden_care_difficult + burden_sleep_problem + b
urden_anxious_attention + burden_exhausted
                  puppyblues =~ burden_care_difficult + burden_concerned_wellbe
ing + burden_irritation_anger + burden_inadequacy
                  + burden_relinquishment + burden_fear_ruin + burden_connect_d
ifficult + burden_exhausted + burden_sleep_problem
                  + burden_anxious_attention + burden_wary + burden_guilt + bur
den_raise_right + burden_cumbersome + burden_regret'
bifactor <- cfa(model_bifactor, data=puppyhood.col,std.lv=TRUE, orthogonal = TR
UE)
summary(bifactor,fit.measures=TRUE,standardized=TRUE)
parameterEstimates(bifactor, standardized = TRUE)

#CFA with a competing one factor revised model (burden_wary removed)
model_1factor_revised <- 'puppyblues =~ burden_care_difficult + burden_concerne
d_wellbeing + burden_irritation_anger + burden_inadequacy
              + burden_relinquishment + burden_fear_ruin + burden_connect_diffi
cult + burden_exhausted
              + burden_sleep_problem + burden_anxious_attention + burden_guilt
+ burden_raise_right
              + burden_cumbersome + burden_regret'
onefactor_revised <- cfa(model_1factor_revised, data=puppyhood.col.revised,std.
lv=TRUE)
summary(onefactor_revised,fit.measures=TRUE,standardized=TRUE)
parameterEstimates(onefactor_revised, standardized = TRUE)

#CFA with a competing two factor revised model (burden_wary removed)
model_2factors_revised <- 'frustration =~ burden_care_difficult + burden_irrita
tion_anger + burden_inadequacy + burden_relinquishment
                  + burden_connect_difficult + burden_exhausted + burden_sleep_
problem + burden_anxious_attention
                  + burden_cumbersome + burden_regret
                  anxiety =~ burden_concerned_wellbeing + burden_inadequacy + b
urden_fear_ruin
                  + burden_guilt + burden_raise_right'
twofactors_revised <- cfa(model_2factors_revised, data=puppyhood.col.revised,st
d.lv=TRUE)
summary(twofactors_revised,fit.measures=TRUE,standardized=TRUE)
parameterEstimates(twofactors_revised, standardized = TRUE)

#CFA with a competing four factor revised model (burden_wary removed)
fourfactormodel_revised <- 'anxiety =~ burden_concerned_wellbeing + burden_inad
equacy + burden_fear_ruin + burden_guilt + burden_raise_right
          frustration =~ burden_irritation_anger + burden_relinquishment
          + burden_connect_difficult + burden_regret
    weariness =~ burden_care_difficult + burden_exhausted + burden_sleep_proble
m + burden_anxious_attention + burden_cumbersome
overwhelm =~ burden_care_difficult + burden_irritation_anger + burden_inadequac
y'
fourfactors_revised <- cfa(fourfactormodel_revised, data=puppyhood.col.revised,
std.lv=TRUE, orthogonal = TRUE)
```

```
summary(fourfactors_revised,fit.measures=TRUE,standardized=TRUE)
parameterEstimates(fourfactors_revised, standardized = TRUE)

#CFA with a competing revised model in which low factor loadings (lv.std<.30) a
re excluded if they load already to another factor
revised_model_3factors <- 'frustration =~ burden_care_difficult + burden_irrita
tion_anger
                  + burden_connect_difficult + burden_cumbersome
  anxiety =~ burden_concerned_wellbeing + burden_inadequacy + burden_fear_ruin
+ burden_wary + burden_guilt + burden_raise_right
  weariness =~ burden_sleep_problem + burden_exhausted'
revised_threefactors <- cfa(revised_model_3factors, data=puppyhood.col,std.lv=T
RUE)
summary(revised_threefactors,fit.measures=TRUE,standardized=TRUE)
parameterEstimates(revised_threefactors, standardized = TRUE)

#CFA with a competing revised (burden_wary removed) bifactor model
bifactormodel_revised <- 'frustration =~ burden_care_difficult + burden_irritat
ion_anger + burden_relinquishment
                  + burden_connect_difficult + burden_cumbersome + burden_regre
t
  anxiety =~ burden_concerned_wellbeing + burden_inadequacy + burden_fear_ruin
+ burden_guilt + burden_raise_right
  weariness =~ burden_care_difficult + + burden_exhausted + burden_sleep_proble
m + burden_anxious_attention + burden_cumbersome
  puppyblues =~ burden_care_difficult + burden_concerned_wellbeing + burden_irr
itation_anger +
  burden_inadequacy + burden_relinquishment + burden_fear_ruin + burden_connect
_difficult +
  burden_exhausted + burden_sleep_problem + burden_anxious_attention + burden_g
uilt +
  burden_raise_right + burden_cumbersome + burden_regret'
bifactor_revised <- cfa(bifactormodel_revised, data=puppyhood.col.revised,std.l
v=TRUE, orthogonal = TRUE)
summary(bifactor_revised,fit.measures=TRUE,standardized=TRUE)
parameterEstimates(bifactor_revised, standardized = TRUE)

#### TEST-RETEST RELIABILITY ####

#Read in the retest data
puppyblues.col.retest <- read_table ()

#Select the original responses of the test-retest participants.
Retest_vastaajat <- read_table ()
puppyblues_test <- left_join(Retest_vastaajat, puppyblues.scores.revised.2, by
= "id")
#Select id, survey items and factor scores
puppyblues.col.test <- puppyblues_test [,c(1,14:28,32:34)]

#Ddd time variables
puppyblues.col.test.time <- puppyblues.col.test %>%
  mutate(time=1)
puppyblues.col.test.time1 <- puppyblues.col.test.time [,c(1:16,20)]
```

```
puppyblues.col.retest.time <- puppyblues.col.retest %>%
  mutate(time=2)
#Join the rows
puppyblues.testretest <- rbind(puppyblues.col.test.time1, puppyblues.col.retest
.time)
#Pearson correlations
keys.list <- list(frustration=c("burden_care_difficult", "burden_irritation_ang
er", "burden_inadequacy",
                                "burden_relinquishment", "burden_connect_diffic
ult", "burden_exhausted",
                                "burden_cumbersome", "burden_regret"),
                  anxiety=c("burden_concerned_wellbeing", "burden_inadequacy",
"burden_fear_ruin", "burden_guilt", "burden_raise_right"),
                  weariness= c("burden_care_difficult", "burden_sleep_problem",
"burden_anxious_attention", "burden_exhausted"))


frustration.testretest <- testRetest(puppyblues.testretest,keys= keys.list$frus
tration, id="id", time="time",check.keys=T, warnings=T,lmer=F)
print(frustration.testretest,short=F)

anxiety.testretest <- testRetest(puppyblues.testretest,keys= keys.list$anxiety,
id="id", time="time",check.keys=T, warnings=T,lmer=F)
print(anxiety.testretest,short=F)

weariness.testretest <- testRetest(puppyblues.testretest,keys= keys.list$wearin
ess, id="id", time="time",check.keys=T, warnings=T,lmer=F)
print(weariness.testretest,short=F)

#Intraclass correlations
#Use data with factor scores (puppyblues.col.test.time)
#Predict factor scores for retest data

#Include only columns that were included in the original
puppyblues.col.retest.time2 <- puppyblues.col.retest.time %>%
  dplyr::select(!c(id,time,burden_wary))

puppyblues.col.test.time2 <- puppyblues.col.test.time %>%
  dplyr::select(!c(id,time,burden_wary))

# Predict factor scores for new data
pupblues.trt.fa <- predict(puppyblues_3_revised, puppyblues.col.retest.time2, p
uppyblues.col.revised)
pupblues.fa <- cbind(puppyblues.col.retest.time, pupblues.trt.fa)

names(pupblues.fa)[names(pupblues.fa) == "ML2"] <- "puppyblues_anxiety"
names(pupblues.fa)[names(pupblues.fa) == "ML1"] <- "puppyblues_frustration"
names(pupblues.fa)[names(pupblues.fa) == "ML3"] <- "puppyblues_weariness"

# Join test and retest datasets
puppyblues.testretest2 <- rbind(puppyblues.col.test.time, pupblues.fa)

# Change to wide format
pupblues.wide <- reshape(puppyblues.testretest, idvar="id",timevar="time",direc
```

```
tion="wide")

### ICC(2,1) scores
#### For loop

# List that will contain all ICC result objects
all_results <- list()

# Data frame that will contain all ICC2,1 values
all_icc <- data.frame("", "", "")
colnames(all_icc) <- c("variable_name","ICC2")
all_icc <- all_icc[-1,]

### i in 2:20: 2 is the number of the first behavior item column
### 20 is the number of the last behavior item of the FIRST respondent
### personality_wide[,c(i,i+19)]: i is again the first behavior item column
### i+19 is the first item of the SECOND respondent: so i and i+19 should be th
e same item
for(i in 2:20) {
  result <- ICC(pupblues.wide[,c(i,i+19)],missing=F,alpha=.05,lmer=F,check.keys
=F)
  variable_name <- colnames(pupblues.wide)[[i]]
  print(variable_name)
  all_results[[variable_name]] <- result
  new_row <- data.frame(variable_name,result$results[2,2])
  colnames(new_row) <- c("variable_name","ICC(2,1)")
  all_icc <- rbind(all_icc, new_row)
}

## View the ICC values for all items and factors
## Paste the table into excel
all_icc

#### VALIDITY ####

#### Predictive validity - polyserial correlations ####
polyserial_anxiety <- polyserial(puppyblues.scores.revised.2$puppyblues_anxiety
, puppyblues.scores.revised.2$subjective_burden)
poly.anxiety.pvalue <- cor.test(puppyblues.scores.revised.2$puppyblues_anxiety,
puppyblues.scores.revised.2$subjective_burden)$p.value
df_anxiety <- length(puppyblues.scores.revised.2$puppyblues_anxiety) - 2
print(paste0("Degrees of Freedom: ", df_anxiety))
print(paste0("Polyserial Correlation: ", polyserial_anxiety))
print(paste0("Significance level: ", poly.anxiety.pvalue))

polyserial_frustration <- polyserial(puppyblues.scores.revised.2$puppyblues_fru
stration, puppyblues.scores.revised.2$subjective_burden)
poly.frustration.pvalue <- cor.test(puppyblues.scores.revised.2$puppyblues_frus
tration, puppyblues.scores.revised.2$subjective_burden)$p.value
df_frustration <- length(puppyblues.scores.revised.2$puppyblues_frustration) -
2
print(paste0("Degrees of Freedom: ", df_frustration))
print(paste0("Polyserial Correlation: ", polyserial_frustration))
print(paste0("Significance level: ", poly.frustration.pvalue))
```

```
polyserial_weariness <- polyserial(puppyblues.scores.revised.2$puppyblues_weari
ness, puppyblues.scores.revised.2$subjective_burden)
poly.weariness.pvalue <- cor.test(puppyblues.scores.revised.2$puppyblues_wearin
ess, puppyblues.scores.revised.2$subjective_burden)$p.value
df_weariness <- length(puppyblues.scores.revised.2$puppyblues_weariness) - 2
print(paste0("Degrees of Freedom: ", df_weariness))
print(paste0("Polyserial Correlation: ", polyserial_weariness))
print(paste0("Significance level: ", poly.weariness.pvalue))

#Table of the results
table_polyserial <- data.frame(
  "polyserial correlation" = c(polyserial_frustration, polyserial_anxiety, poly
serial_weariness),
  "p-value" = c(poly.frustration.pvalue, poly.anxiety.pvalue, poly.weariness.pv
alue),
  row.names = c("frustration", "anxiety", "weariness"))
print(table_polyserial)

#### Predictive validity - box plots ####

puppyblues.scores.revised.2$subjective_burden <- as.factor(puppyblues.scores.re
vised.2$subjective_burden)

anxiety_burden <- ggplot(data=puppyblues.scores.revised.2, aes(y=puppyblues_anx
iety,x=subjective_burden)) + geom_boxplot() +
  labs(title="Puppy Blues Anxiety - Subjective Burden",y="Puppy Blues Anxiety S
core",
       x="Subjective Burden") +
  theme(axis.title=element_text(size=13),axis.text=element_text(size=12),
        axis.line=element_line(linewidth=0.5),
        plot.background = element_rect(fill="white"),
        panel.background = element_rect(fill="white"),
        panel.grid=element_blank())

frustration_burden <- ggplot(data=puppyblues.scores.revised.2, aes(y=puppyblues
_frustration,x=subjective_burden)) + geom_boxplot() +
  labs(title="Puppy Blues Frustration - Subjective Burden",y="Puppy Blues Frust
ration Score",
       x="Subjective Burden") +
  theme(axis.title=element_text(size=13),axis.text=element_text(size=12),
        axis.line=element_line(linewidth=0.5),
        plot.background = element_rect(fill="white"),
        panel.background = element_rect(fill="white"),
        panel.grid=element_blank())

weariness_burden <- ggplot(data=puppyblues.scores.revised.2, aes(y=puppyblues_w
eariness,x=subjective_burden)) + geom_boxplot() +
  labs(title="Puppy Blues Weariness - Subjective Burden",y="Puppy Blues Wearine
ss Score",
       x="Subjective Burden") +
  theme(axis.title=element_text(size=13),axis.text=element_text(size=12),
        axis.line=element_line(linewidth=0.5),
        plot.background = element_rect(fill="white"),
```

```r
        panel.background = element_rect(fill="white"),
        panel.grid=element_blank())

#Arrange plot on the same page
PB_factors.burden <- ggarrange(anxiety_burden, frustration_burden, weariness_bu
rden,
                              ncol=1, nrow=3,
                              common.legend = TRUE, legend = "bottom",
                              labels = c("A", "B", "C"), font.label = list(siz
e = 14))

#Save PDF file
ggsave(filename="PB_factors.burden.pdf",PB_factors.burden,device="pdf",
       width=2000, height=4000,units="px")

#### Cross-validity ####

#Set random seed for reproducibility
set.seed(123)

#Select id and items
puppyblues.cross <- puppyblues[,c(1,13:22,24:27)]

#Create training and testing indices using caret::createFolds
folds <- createFolds(puppyblues.cross$id, k = 2, returnTrain = TRUE)

#Extract the training and testing data sets
train_data <- puppyblues.cross[folds$Fold1, ]
test_data <- puppyblues.cross[folds$Fold2, ]

#Conduct exploratory factor analysis on training data
efa_train_data <- fa(train_data[,c(2:15)], nfactors=3, scores="Thurstone", warn
ings=TRUE, fm="ml", cor="poly", missing=T, impute="mean")

#Print factor loadings for the EFA model
print(efa_train_data, cut=0.3)

print(efa_train_data)

#Test the factor solution on the testing data
#Specify the CFA model based on the EFA results
cfa_cross <- "
  Anxiety =~ burden_concerned_wellbeing + burden_inadequacy + burden_fear_ruin
+ burden_guilt
  + burden_raise_right + burden_cumbersome
  Weariness =~ burden_care_difficult + burden_exhausted + burden_sleep_problem
+ burden_anxious_attention + burden_cumbersome
  Frustration =~ burden_irritation_anger + burden_relinquishment + burden_conne
ct_difficult + burden_regret
"

#Fit the CFA model to the testing data
CFA_cross3 <- cfa(cfa_cross, data=test_data,std.lv=TRUE)
```

```
#Print the standardized factor loadings from the CFA model
summary(CFA_cross3,fit.measures=TRUE,standardized=TRUE)

#### construct validity - Comparison of scores related to the dog's puppyhood a
nd adulthood (1-2 years) ####

#T-test: is the burden lower when the dog is 1-2-year-old compared to puppyhood
?
t_test_result <- t.test(puppy_adult$subjective_burden_v2, puppy_adult$subjectiv
e_burden, paired = TRUE)

print(t_test_result)

#Calculate factor scores for the data
#Select puppy blues items
puppyhood.col <- puppy_adult[,c(6:15,17:20)]
adulthood.col <- puppy_adult[,c(54:63,65:68)]

#Predict values for puppy blues
puppyblues_fa <- predict(puppyblues_3_revised, puppyhood.col, puppyblues.col.re
vised)

puppy_adult_factorscores <- cbind(puppy_adult, puppyblues_fa)
names(puppy_adult_factorscores)[names(puppy_adult_factorscores) == "ML1"] <- "p
uppyblues_anxiety"
names(puppy_adult_factorscores)[names(puppy_adult_factorscores) == "ML2"] <- "p
uppyblues_frustration"
names(puppy_adult_factorscores)[names(puppy_adult_factorscores) == "ML3"] <- "p
uppyblues_weariness"

#Predict values for current burden
adultblues_fa <- predict(puppyblues_3_revised, adulthood.col, puppyblues.col.re
vised)

puppy_adult_factorscores_v2 <- cbind(puppy_adult_factorscores, adultblues_fa)
names(puppy_adult_factorscores_v2)[names(puppy_adult_factorscores_v2) == "ML1"]
<- "puppyblues_anxiety_v2"
names(puppy_adult_factorscores_v2)[names(puppy_adult_factorscores_v2) == "ML2"]
<- "puppyblues_frustration_v2"
names(puppy_adult_factorscores_v2)[names(puppy_adult_factorscores_v2) == "ML3"]
<- "puppyblues_weariness_v2"

#T-test: are the anxiety scores lower now compared to puppyhood?
t_test_anxiety <- t.test(puppy_adult_factorscores_v2$puppyblues_anxiety_v2, pup
py_adult_factorscores_v2$puppyblues_anxiety, paired = TRUE)
print(t_test_anxiety)

#T-test: are the frustration scores lower now compared to puppyhood?
t_test_frustration <- t.test(puppy_adult_factorscores_v2$puppyblues_frustration
_v2, puppy_adult_factorscores_v2$puppyblues_frustration, paired = TRUE)
print(t_test_frustration)

#T-test: are the weariness scores lower now compared to puppyhood?
t_test_weariness <- t.test(puppy_adult_factorscores_v2$puppyblues_weariness_v2,
```

```
puppy_adult_factorscores_v2$puppyblues_weariness, paired = TRUE)
print(t_test_weariness)

#Box plots of the difference in factor scores
boxplot_frustraton <- ggplot(puppy_adult_factorscores_v2, aes(x = factor(1), y
= puppyblues_frustration)) +
  geom_boxplot() +
  geom_boxplot(aes(x = factor(2), y = puppyblues_frustration_v2)) +
  scale_y_continuous(limits = c(-1.5, 10)) +
  xlab("") +
  scale_x_discrete(labels = c("Puppy", "1-2-year-old dog")) +
  ylab("Puppy Blues Frustration") +
  theme(axis.title=element_text(size=16),axis.text=element_text(size=14),
        axis.line=element_line(linewidth=0.5),
        plot.background = element_rect(fill="white"),
        panel.background = element_rect(fill="white"),
        panel.grid=element_blank()) +
  geom_segment(aes(x = 1, xend = 2, y = 7, yend = 7), linetype = "dashed") +
  geom_segment(aes(x = 1, xend = 1, y = 6.9, yend = 7.1))  +
  geom_segment(aes(x = 2, xend = 2, y = 6.9, yend = 7.1)) +
  annotate("text", x = 1.5, y = 7.4, label = "p < 0.001", size = 4)

boxplot_anxiety <- ggplot(puppy_adult_factorscores_v2, aes(x = factor(1), y = p
uppyblues_anxiety)) +
  geom_boxplot() +
  geom_boxplot(aes(x = factor(2), y = puppyblues_anxiety_v2)) +
  scale_y_continuous(limits = c(-1.5, 10)) +
  xlab("") +
  scale_x_discrete(labels = c("Puppy", "1-2-year-old dog")) +
  ylab("Puppy Blues Anxiety") +
  theme(axis.title=element_text(size=16),axis.text=element_text(size=14),
        axis.line=element_line(linewidth=0.5),
        plot.background = element_rect(fill="white"),
        panel.background = element_rect(fill="white"),
        panel.grid=element_blank()) +
  geom_segment(aes(x = 1, xend = 2, y = 9, yend = 9), linetype = "dashed") +
  geom_segment(aes(x = 1, xend = 1, y = 8.9, yend = 9.1)) +
  geom_segment(aes(x = 2, xend = 2, y = 8.9, yend = 9.1)) +
  annotate("text", x = 1.5, y = 9.4, label = "p < 0.001", size = 4)


boxplot_weariness <- ggplot(puppy_adult_factorscores_v2, aes(x = factor(1), y =
puppyblues_weariness)) +
  geom_boxplot() +
  geom_boxplot(aes(x = factor(2), y = puppyblues_weariness_v2)) +
  scale_y_continuous(limits = c(-1.5, 10)) +
  xlab("") +
  scale_x_discrete(labels = c("Puppy", "1-2-year-old dog")) +
  ylab("Puppy Blues Weariness") +
  theme(axis.title=element_text(size=16),axis.text=element_text(size=14),
        axis.line=element_line(linewidth=0.5),
        plot.background = element_rect(fill="white"),
        panel.background = element_rect(fill="white"),
        panel.grid=element_blank()) +
```

```r
  geom_segment(aes(x = 1, xend = 2, y = 8, yend = 8), linetype = "dashed") +
  geom_segment(aes(x = 1, xend = 1, y = 7.9, yend = 8.1)) +
  geom_segment(aes(x = 2, xend = 2, y = 7.9, yend = 8.1)) +
  annotate("text", x = 1.5, y = 8.4, label = "p < 0.001", size = 4)

boxplot_factors <- ggarrange(boxplot_anxiety, boxplot_frustraton, boxplot_weari
ness,
                             ncol=3, nrow=1,
                             common.legend = TRUE, legend = "bottom",
                             labels = c("a", "b", "c"), font.label = list(size
= 14))

#Save PDF
ggsave(filename = "Figure2.jpeg",
       plot = boxplot_factors,
       device = "jpeg",
       width = 15,
       height = 6,
       units = "in",
       dpi = 300)


#frequencies of increase/decrease in factor scores

# define SD for anxiety factor scores
sd(puppy_adult_factorscores_v2$puppyblues_anxiety, na.rm = TRUE)
sd_diff_anxiety <- c(-Inf, -2.054966, -1.027483, 0, 1.027483, 2.054966, Inf)

puppy_adult_factorscores_v2 <- puppy_adult_factorscores_v2 %>%
  mutate(diff_anxiety = as.numeric(puppyblues_anxiety_v2 - puppyblues_anxiety))

sd_freq_anxiety <- table(cut(puppy_adult_factorscores_v2$diff_anxiety, breaks =
sd_diff_anxiety, labels = c("< -1 SD", "-1 to -0.5 SD", "-0.5 to 0 SD", "0 to 0
.5 SD", "0.5 to 1 SD", "> 1 SD")), useNA = "always")
print(sd_freq_anxiety)

# define SD for frustration factor scores
sd(puppy_adult_factorscores_v2$puppyblues_frustration, na.rm = TRUE)
sd_diff_frustration <- c(-Inf, -1.58175, -0.790875, 0, 0.790875, 1.58175, Inf)

puppy_adult_factorscores_v2 <- puppy_adult_factorscores_v2 %>%
  mutate(diff_frustration= as.numeric(puppyblues_frustration_v2 - puppyblues_fr
ustration))

sd_freq_frustration <- table(cut(puppy_adult_factorscores_v2$diff_frustration,
breaks = sd_diff_frustration, labels = c("< -1 SD", "-1 to -0.5 SD", "-0.5 to 0
SD", "0 to 0.5 SD", "0.5 to 1 SD", "> 1 SD")), useNA = "always")
print(sd_freq_frustration)

# define SD for weariness factor scores
sd(puppy_adult_factorscores_v2$puppyblues_weariness, na.rm = TRUE)
sd_diff_weariness <- c(-Inf, -1.807574, -0.903787, 0, 0.903787, 1.807574, Inf)

puppy_adult_factorscores_v2 <- puppy_adult_factorscores_v2 %>%
```

```
  mutate(diff_weariness= as.numeric(puppyblues_weariness_v2 - puppyblues_wearin
ess))

sd_freq_weariness <- table(cut(puppy_adult_factorscores_v2$diff_weariness, brea
ks = sd_diff_weariness, labels = c("< -1 SD", "-1 to -0.5 SD", "-0.5 to 0 SD",
"0 to 0.5 SD", "0.5 to 1 SD", "> 1 SD")), useNA = "always")
print(sd_freq_weariness)

#### fading affect bias - scatter plots ####

plot1 <- ggplot(data=puppyblues.scores.revised.2, aes(y=puppyblues_frustration,
x=timefrombirth_years)) +
  geom_point() +
  labs(title="Puppy Blues Frustration - Time from Birth",y="Puppy Blues Frustra
tion Score",x="Time from Birth (years)") +
  theme(axis.title=element_text(size=13),axis.text=element_text(size=12),
        axis.line=element_line(linewidth=0.5),
        plot.background = element_rect(fill="white"),
        panel.background = element_rect(fill="white"),
        panel.grid=element_blank()) +
  coord_cartesian(ylim=c(-2,8),xlim=c(2,21),expand=F)

plot2 <- ggplot(data=puppyblues.scores.revised.2, aes(y=puppyblues_anxiety,x=ti
mefrombirth_years)) +
  geom_point() +
  labs(title="Puppy Blues Anxiety - Time from Birth",y="Puppy Blues Anxiety Sco
re",x="Time from Birth (years)") +
  theme(axis.title=element_text(size=13),axis.text=element_text(size=12),
        axis.line=element_line(linewidth=0.5),
        plot.background = element_rect(fill="white"),
        panel.background = element_rect(fill="white"),
        panel.grid=element_blank()) +
  coord_cartesian(ylim=c(-2,8),xlim=c(2,21),expand=F)


plot3 <- ggplot(data=puppyblues.scores.revised.2, aes(y=puppyblues_weariness,x=
timefrombirth_years)) +
  geom_point() +
  labs(title="Puppy Blues Weariness - Time from Birth",y="Puppy Blues Weariness
Score",x="Time from Birth (years)") +
  theme(axis.title=element_text(size=13),axis.text=element_text(size=12),
        axis.line=element_line(linewidth=0.5),
        plot.background = element_rect(fill="white"),
        panel.background = element_rect(fill="white"),
        panel.grid=element_blank()) +
  coord_cartesian(ylim=c(-2,8),xlim=c(2,21),expand=F)

#Arrange plots on the same page
fading_affect <- plot1 + plot2 + plot3 +
  plot_layout(ncol=1, nrow=3) + plot_annotation(tag_levels="A")

#Save PDF
ggsave(filename="fading_affect.pdf",fading_affect,device="pdf",
       width=2000, height=4000,units="px")
```

```
#### Convergent validity ####
hypothesis_columns <- puppyblues.scores.revised.2[, c("puppyblues_frustration",
                                                      "puppyblues_anxiety", "pu
ppyblues_weariness",
                                                      "PAQ_Avoidance", "PAQ_Anx
iety", "NeuroticismM",
                                                      "CESD.10", "GAD.7")]
cor_results <- corr.test(hypothesis_columns,
                         method = "pearson", adjust = "fdr", alpha = 0.001)
print(cor_results$r)
print(cor_results$p)
```
